# Quantitative proteomics analysis of lysine 2-hydroxyisobutyrylation in IgA nephropathy

**DOI:** 10.1186/s12014-021-09314-0

**Published:** 2021-02-08

**Authors:** Shaoying Huang, Fengping Zheng, Hua Lin, Xianqing Zhou, Huixuan Xu, Cantong Zhang, Weier Dai, Berthold Hocher, Xinzhou Zhang, Donge Tang, Yong Dai

**Affiliations:** 1 Department of Clinical Medical Research Center, Guangdong Provincial Engineering Research Center of Autoimmune Disease Precision Medicine, The Second Clinical Medical College of Jinan University, The First Affiliated Hospital Southern University of Science and Technology, Shenzhen People’s Hospital, Shenzhen, 518020 Guangdong People’s Republic of China; 2 Department of Nephrology, the Second Clinical Medical College of Jinan University, the First Affiliated Hospital of Southern University of Science and Technology, Shenzhen People’s Hospital, Shenzhen, 518020 People’s Republic of China; 3 Guangxi Key Laboratory of Metabolic Disease Research, Nephrology Department of Guilin NO. 924 Hospital, Guilin, 541002 People’s Republic of China; 4 College of Natural Science, University of Texas at Austin, Austin, Texas 78712 USA; 5 Department of Medicine Nephrology, Medical Faculty Mannheim Heidelberg University, Mannheim, Germany

**Keywords:** Lysine 2-hydroxyisobutyrylation, Mass spectrometry, Immunoglobulin A nephropathy, Peripheral blood mononuclear cells

## Abstract

**Background:**

Protein posttranslational modification is an indispensable regulatory element that can fine-tune protein functions and regulate diverse cellular processes. Lysine 2-hydroxyisobutyrylation (Khib) is a protein posttranslational modification that was recently identified and is thought to play a role in a wide variety of active cellular functions.

**Methods:**

In this report, for the first time, we comparatively studied the 2-hydroxyisobutyrylation proteome in peripheral blood mononuclear cells from a biopsy-proven immunoglobulin A nephropathy (IgAN) group and a normal control group based on liquid chromatography-tandem mass spectrometry.

**Results:**

Altogether, 7405 proteins were identified and added to a Khib library. Of these proteins, we identified 111 with upregulated expression and 83 with downregulated expression. Furthermore, we identified 428 Khib modification sites on 290 Khib-modified proteins, including 171 sites with increased modification on 122 Khib-modified proteins and 257 specific sites with reduced modification on 168 Khib-modified proteins.

**Conclusions:**

Importantly, the abundance of lipocalin 2 was increased in the differentially expressed proteins, and a KEGG-based functional enrichment analysis showed that Khib proteins clustered in the IL-17 signaling pathway and phagosome category, which may have important associations with IgAN. Our data enlighten our understanding of Khib in IgAN and indicate that Khib may have important regulatory roles in IgAN.

## Introduction

Protein posttranslational modification (PTMs) are competing processes involved in the coordination and regulation of protein properties and activities at the molecular level, such as stability, interactions, cellular localization and enzymatic activity [1–4]. Additionally, PTMs may be inserted between the regulatory enzymes and the modified substrates affecting protein functions through the influence of internucleosomal communication or charge state [5, 6].

Among the protein family of PTMs, there are hundreds of novel lysine acylation modifications, such as 2-hydroxyisobutyrylation, glutarylation, propionylation, succinylation, and malonylation, that have been discovered throughout the last few years. It has been suggested that cellular metabolism is closely associated with epigenetic mechanisms [7, 8]. The lysine 2-hydroxyisobutyrylation (Khib) pathway, therefore, provides another opportunity to influence and alter epigenetic networks at the cellular level.

Khib is a recently identified protein lysine acylation PTM that is evolutionarily conserved across plants, *Proteus mirabilis*, mice and humans [9–12]. 2-Hydroxyisobutyrylated proteins are generally involved in transcription, translation, and protein degradation and have been shown to be enriched in metabolic pathways biologically through bioinformatics analyses [13]. It has been indicated that Khib acts in vital cellular processes and networks, preferentially including glycolysis/gluconeogenesis, and the TCA cycle, and was shown to be involved in protein biosynthesis and processing in a study describing rice seeds [10]. Khib shares the same sites as other forms of lysine acylation not only on histones but also on non-histone proteins [10].

Immunoglobulin A nephropathy (IgAN) is a regular pathologic feature of primary glomerulonephritis among patients with renal biopsy worldwide and causes a burden to society in the form of chronic and end-stage kidney disease [14, 15]. Currently, there are distinct differences in IgAN prevalence based on ethnicity and race, but the incidence rate is increased among Asian populations [16]. Clinically, the current standardized protocol for the diagnosis of IgAN is the histological scoring of lesion samples collected by renal biopsy [17], which is, however, invasive and imposes a physical challenge and attendant risk to patients. No genetic or serologic test, to date, can reliably distinguish IgAN from other diseases. Recently, peripheral blood mononuclear cells (PBMCs) have attracted increasing attention as objects of proteomic profiling studies. Instead of a biopsy, PBMCs can be conveniently isolated from peripheral blood and are mainly composed of monocytes, dendritic cells and lymphocytes. The pathogenic mechanisms of IgAN remain largely unknown. It is acknowledged that a major pathologic contributor to the risk of disease is high levels of IgA in the peripheral blood, particularly in a form that is relatively galactose deficient [18]. Galactose-deficient O-glycan IgA1 (Gd-IgA1) is considered a biomarker for IgAN and is associated with glycosylation, which is a PTM similar to Khib. Since PBMCs are involved in IgAN pathogenesis [19–21], we sought to explore the Khib proteomic profiles of PBMCs isolated from IgAN patients.

## Materials and methods

### Materials

Trypsin and formic acid were obtained from Fluka (Buches, Germany) and Promega (Fitchburg, WI), respectively. Iodoacetamide, dithiothreitol, urea, EDTA, NAM and TSA were purchased from Sigma (St. Louis, MO). A TMT kit was obtained from Thermo (Waltham, MA). Pure water and acetonitrile were obtained from Fisher Chemical. Trifluoroacetic acid was purchased from Sigma-Aldrich. Protease inhibitors were obtained from Calbiochem. A BCA kit was obtained from Beyotime.

### Patients and control subjects

The PBMC samples were retrieved from patients with biopsy-diagnosed IgAN at the Shenzhen People’s Hospital before any kind of treatment initiation. Moreover, all patients with other obvious complications or severe primary diseases, such as cardiovascular, anaphylactoid purpura, liver and kidney diseases, were excluded.

A total of 5 patients (aged between 20 and 50 years) initially diagnosed with IgAN at the Shenzhen People’s Hospital from October 2017 to October 2018 were recruited (Table 1). In addition, the normal control (NC) group (aged between 20 and 50 years) was enrolled from patients at the Physical Examination Centre at the Shenzhen People’s Hospital for routine health examination without any disease. All of the research volunteers were from southern China. After sample collection, cells were isolated by Ficoll density gradient centrifugation and then stored at − 80 °C.

**Table 1:** Basic characteristics of 5 patients with IgAN

	Patient 1	Patient 2	Patient 3	Patient 4	Patient 5
Sex (male/female)	Male	Female	Female	Male	Male
Age (years)	29	33	19	48	23
eGFR(ml/min/1.73 m^2^)	95	43	129	3	140
Creatinine (μmol/L)	93	138	57	1610	70
UA (μmol/L)	273	283	204	619	393
24-h urinary protein (g/24 h)	0.72	1.5	NA	1.7	0.36
Hematuria	Negative	Negative	Negative	Negative	Negative
Oxford classification					
M 0/1	1	1	1	1	1
E 0/1	0	0	0	0	0
S 0/1	0	0	0	1	1
T 0/1/2	0	0	0	0	0
C 0/1/2	0	0	0	1	0

### Protein extraction

PBMC lysis was performed in ice-cold lysis buffer (containing 8 mol/L urea, 1% protease inhibitors, 3 μmol/L TSA, and 50 mmol/L NAM) with a high-intensity ultrasonic processor instrument (PTM Bio, Hangzhou, China), after which the residual cell components were removed immediately by centrifugation (12,000*g* at 4 °C for 10 min). Afterward, the supernatant was transferred to a new tube for further centrifugation. First, the cellular debris of the serum sample was removed immediately by centrifugation (12,000*g* at 4 °C for 10 min). Then, the supernatant was moved to a new centrifuge tube. Finally, the protein concentration was defined by a BCA kit according to the manufacturer’s instructions.

### Trypsin digestion

The protein solution was sequentially diluted (5 mmol/L dithiothreitol for 30 min at 56 °C) and alkylated with 11 mmol/L iodoacetamide for 15 min. The above procedures were performed in darkness at room temperature. Then, the assembled protein sample was diluted by adding a total of 100 mmol/L TEAB to a urea concentration of less than 2 mol/L. Finally, trypsin was added to begin (the ratio of trypsin to protein mass ratio was 1:50) all-night digestion and a subsequent (the ratio of trypsin to protein mass was 1:100) 4 h-digestion.

### TMT labeling

The peptides were desalted by a Strata X C18 SPE column from Phenomenex. They were vacuum-dried and reconstituted in 0.5 mol/L TEAB and then processed for the TMT kit. Briefly, one unit of thawed TMT reagent was reconstituted in acetonitrile. Next, the obtained mixtures were incubated (2 h at room temperature), and they were further pooled, desalted and dried by vacuum centrifugation.

### HPLC fractionation

The tryptic peptides were fractionated by high pH reverse-phase HPLC using a Thermo BetaSil C18 column (5 μm particles, 10 mm ID, 250 mm length). Briefly, peptides were divided into 60 fractions with a gradient of 8 to 32% acetonitrile (pH 9.0) for over 60 min, combined in a single fraction and then dried by vacuum centrifugation.

### Affinity enrichment of lysine 2-hydroxyisobutyrylated peptides

To enrich peptides modified by 2-hydroxyisobutyrylation, tryptic peptides dissolved in IP buffer (pH 8.0, 100 mmol/L NaCl, 50 mmol/L Tris–HCl, 1 mmol/L EDTA, 0.5% NP-40) were first incubated with prewashed antibody beads (PTM804, PTM Bio) at 4 °C overnight with gentle shaking. Then, they were washed four times with IP buffer and twice with H_2_O. Afterward, peptides were eluted from the beads with 0.1% trifluoroacetic acid. The fractions were eluted and then combined in a vacuum dryer. Finally, the resulting peptides were desalted by C18 ZipTips (Millipore) for the subsequent analysis.

### LC–MS/MS analysis

The tryptic peptides were dissolved in an aqueous solution containing 0.1% formic acid and 2% acetonitrile (solvent A) and straight loaded into a reversed-phase domestic analytical column (15 cm length, and 75 μm). For the Khib proteomics, the liquid phase gradient consisted of an increase from 5 to 18% solvent B (0.1% formic acid in 90% acetonitrile) for 38 min, 18 to 32% over the next 38 to 52 min and rose to 80% from 52 to 56 min, all maintained at a constant flow rate of 400 nL/min for 56 to 60 min on an EASY-nLC 1000 UPLC system. The peptides underwent MS/MS in Q Exactive™ Plus (Thermo) linked to an online UPLC immediately after the NSI source (electrospray voltage: 2.0 kV). The m/z scan range was between 350 and 1800 to run a comprehensive scan. Intact peptides were then searched and detected in an Orbitrap ion trap mass analyzer at an unalterable resolution of 70,000. The peptides selected for MS/MS were set at a fixed starting point m/z scan of 100, and the fragments were probed in the Orbitrap at a resolution of 35,000. A data-dependent mode that could alternate between one MS scan and 20 scans was applied with 15.0 s dynamic exclusion. The maximum injection time was set to 200 ms. Automatic gain control (AGC) was set at 5E4, and the signal threshold was set to 10,000 ions/s for the whole-cell proteomics and 5000 ions/s for the Khib analysis.

### Database search

The MaxQuant search engine (v.1.5.2.8) was used to process the resulting data. MS were examined using the SwissProt Human database appended with a reverse decoy database. Common contamination libraries were added to offset the impact of contaminating proteins in the identification results. Trypsin/P, a cleavage enzyme, allowed up to 2 missing cleavages for the whole-cell proteomics and 4 missing cleavages for the Khib analysis. The mass tolerance was adjusted to a parameter of 20 ppm at the beginning and 5 ppm for the next major search for precursor ions; the mass tolerance for fragment ions was set as 0.02 Da. The false-positive discovery rate (FDR) was adjusted to < 0.01. The minimal score was set as > 40 for modified peptides. For the whole-cell proteomics, the fixed modification was carbamidomethyl on Cys, and the variable modifications were oxidation on Met and acetylation of the N-terminus of proteins. In addition, lysine 2-hydroxyisobutyrylation modification, acetylation of the protein N-terminus and oxidation on Met were specified as variable modifications for the 2-hydroxyisobutyrylation.

### PRM analysis

The results of the Khib proteomics were used to select proteins for PRM validation in this study. Preparations, including protein extraction and trypsin digestion described in previous procedures, were conducted at the initiation of PRM analysis. The liquid phase gradient consisted of an increase from 6 to 25% solvent B in the first 40 min, 25% to 35% in the next 40 to 52 min and a rise to 80% from 52 to 56 min, all at a constant flow rate of 400 nL/min for 56 to 60 min on an EASY-nLC 1000 UPLC system. Afterward, the peptides were analyzed by MS/MS in Q Exactive™ Plus combined and connected to the UPLC and then they went through the NSI source with the electrospray voltage preset at 2.0 kV. The range of the m/z scan was adjusted from 320 to 1030 for a full scan, and the resulting peptides were detected by Orbitrap at a resolution setting of 70,000. Afterward, peptides were chosen and applied to MS/MS using the NCE tool at a setting of 27. Fragments detected in the Orbitrap were set at a resolution of 17,500. A data-independent mode was alternated between one MS scan and 20 scans. AGC was set at 3E6 mode for the whole MS, and 1E5 mode was specified for MS/MS. The maximal value of IT was adjusted to 50 ms for a complete MS and 170 ms for MS/MS. The isolation window was set to 1.6 m/z for MS/MS.

The MS data were then analyzed and displayed by Skyline (v.3.6). Peptide settings were presented as follows: the enzyme was set as trypsin[KR/P]. The peptide length of the amino acid residue was set as 7–25. The maximum missed cleavage was set as 0. Cysteine alkylation was set as a fixed modification. Additionally, transition settings were presented as follows: ion charges were set as 1, while precursor charges were set as 2 and 3, and ion types were set as b, y. The product ions were determined over a range from ion 3 to the last ion; furthermore, the ion match tolerance was adjusted to 0.02 Da.

### Bioinformatics methods

#### GO annotation

Gene Ontology (GO) annotation of the proteome was derived from the UniProt-GOA database (http://www.ebi.ac.uk/GOA/). First, the protein IDs identified in this study of IgAN were converted to UniProt IDs, which were mapped to the GO IDs by protein ID. The protein sequence alignment method was used to annotate the protein GO function through InterProScan (a sequence analysis application) in the case of certain identified proteins without annotations in UniProt-GOA. were classified by GO annotation based on three categories as follows: molecular function, biological process and cellular component.

#### Domain annotation

The protein domain functional descriptions identified in this study were annotated by InterProScan, which analyzes the data through both the InterPro domain database (http://www.ebi.ac.uk/interpro/) and the protein sequence alignment.

#### KEGG pathway annotation

First, KAAS, an online service tool from the Kyoto Encyclopedia of Genes and Genomes (KEGG) (http://www.genome.jp/kegg/), was used to annotated proteins according to the KEGG database, which was then used to map the annotation results.

#### Subcellular localization

WoLF PSORT (v.0.2 http://www.genscript.com/psort/wolf_psort.html) was used in our study of IgAN to predict and characterize the subcellular localization of the identified proteins.

#### GO enrichment analysis

For each GO-annotation category, a two-tailed Fisher’s exact test was employed as a statistical significance test to verify the enrichment of the differentially expressed proteins (DEPs) in our study (P value < 0.05).

#### Pathway enrichment analysis

The KEGG database was used in this study to identify enriched pathways and to test the enrichment of the differentially Khib-modified proteins against all identified proteins by a two-tailed Fisher’s exact test. The pathways with corrected P values < 0.05 were considered significant and were separated into individual categories.

#### Enrichment analysis of the protein domains

The InterPro database (a resource that provides functional analyses according to protein sequences) was searched for proteins in each category, and a two-tailed Fisher’s exact test was utilized to examine the enrichment of the differentially Khib-modified proteins compared to all identified Khib-modified proteins. The enrichment of protein domains with a corrected P value < 0.05 was considered significant.

#### Enrichment-based clustering

We first collated all the categories based on the results from both enrichment analyses along with their P values for further clustering analysis depending on the differentially Khib-modified protein functional classification (such as GO, domain and pathway). After that, we picked the categories with at least one enriched cluster and P value < 0.05. The filtered cluster P value was mathematically z-transformed as a function of x =  − log10 (P value) for each functional category. These z scores were further clustered by one-way hierarchical clustering using distance and average linkage clustering in Genesis. A heat map was used to visualize the clusters using the “gplots” R-package.

#### Protein–protein interaction network

All differentially expressed Khib-modified protein accession numbers or sequences were examined thoroughly by the STRING database (version 10.5) for convenient presentation and protein–protein interaction analysis. We excluded external candidates by choosing only the interactions between proteins belonging to the searched data set. A confidence score defined by STRING was applied to assess the interaction confidence; a confidence score > 0.7 was defined as high confidence. Finally, the interaction network was visualized using the R package “networkD3” from STRING.

## Results

### Proteomics screening of PBMCs from IgAN patients and NCs

**Fig. 1:**
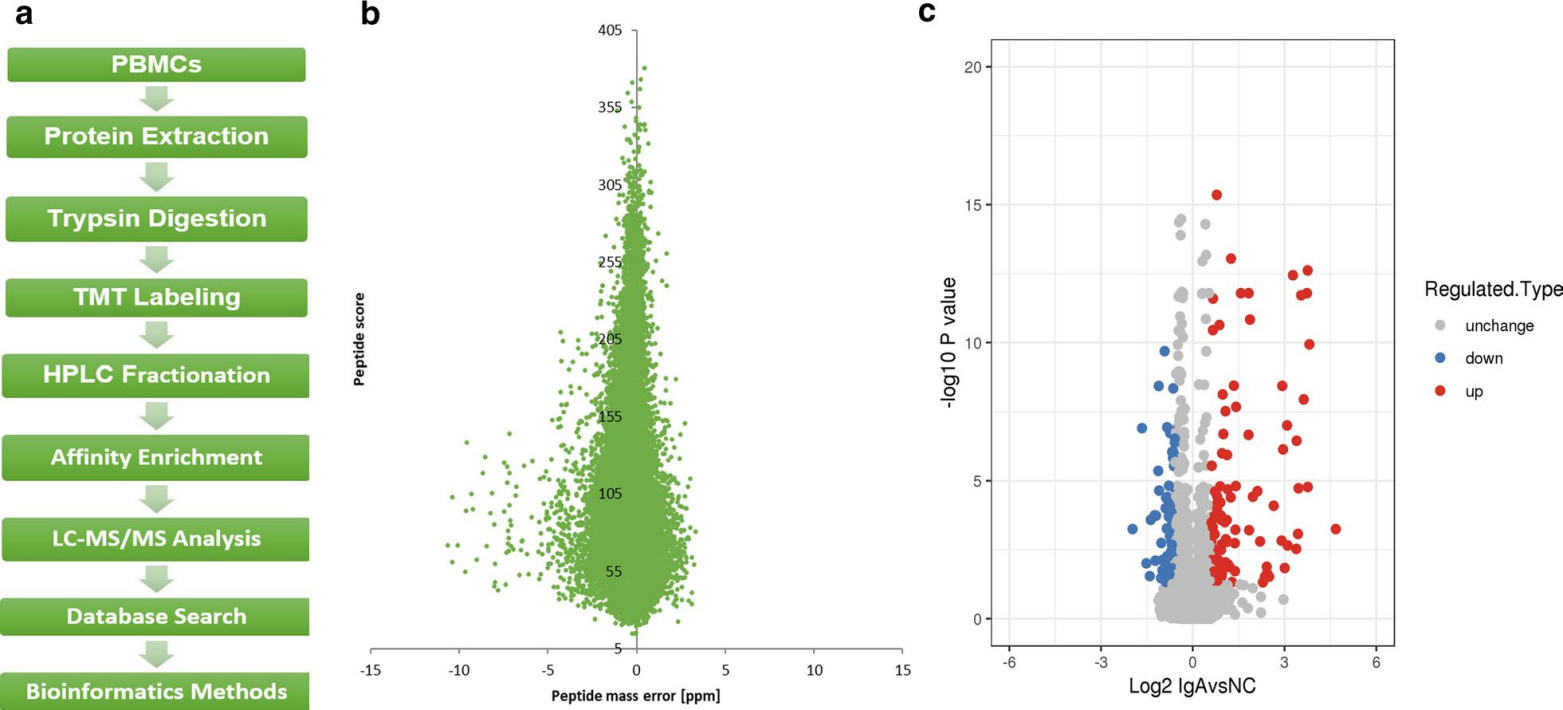
Comparative analysis of whole proteome and Khib in IgAN patients and NC. **a** Overview of experimental procedures. **b** Mass error distribution of all Khib peptides. **c** Volcano plot of the differentially expressed proteins in the IgAN

We carried out the experimental procedure in 9 steps (Fig. 1a) and established a library of proteins differentially expressed between IgAN patients and NCs. Altogether, 6502 DEPs were identified based on 52,362 unique peptides. The confident identification of proteins required an FDR < 0.01 and at least two unique peptides per protein to be present; therefore, 5848 proteins met the above criteria. To check the acquired MS data, we verified that the mass error was between – 11 and 4 ppm, which meets the requirement of mass accuracy (Fig. 1b). Most peptides ranged from 7 to 20 amino acids in length, which is consistent with the basic principle of trypsin digestion. The cutoff for the identification of DEPs or differentially expressed sites (DESs) was a P value < 0.05. A greater than 1.5-fold change was termed upregulation, while a fold change of < 1/1.5 was termed downregulation for DEPs or DESs. Of the DEPs between IgAN patients and NCs, 111 were upregulated and 83 were downregulated (Fig. 1c), including LCN2 (IgA/NC ratio: 13.503, IgA/NC P value < 0.001), LTF (IgA/NC ratio: 13.304, IgA/NC P value < 0.001), MMP9 (IgA/NC ratio: 9.663, IgA/NC P value < 0.001), MPO (IgA/NC ratio: 5.675, IgA/NC P value < 0.001), S100A9 (IgA/NC ratio: 5.663, IgA/NC P value < 0.001), S100A8 (IgA/NC ratio: 5.079, IgA/NC P value < 0.001) and MYH9 (IgA/NC ratio: 1.571, IgA/NC P value < 0.001).

### Functional classification of subcellular localization and GO analysis of the Khib proteome

The subcellular localization of the Khib proteome was illustrated by WoLF PSORT software, and the upregulated 2-hydroxyisobutyrylated proteins were found to be mainly distributed in the extracellular space (35%), cytoplasm (33%), nucleus (12%), plasma membrane (10%), and mitochondria (5%). On the contrary, the majority of the downregulated proteins were distributed in the cytoplasm (40%), nucleus (27%), and extracellular region (18%). A significant difference was not observed in the localization of the upregulated and downregulated proteins based on the data presented in our study.

**Fig. 2:**
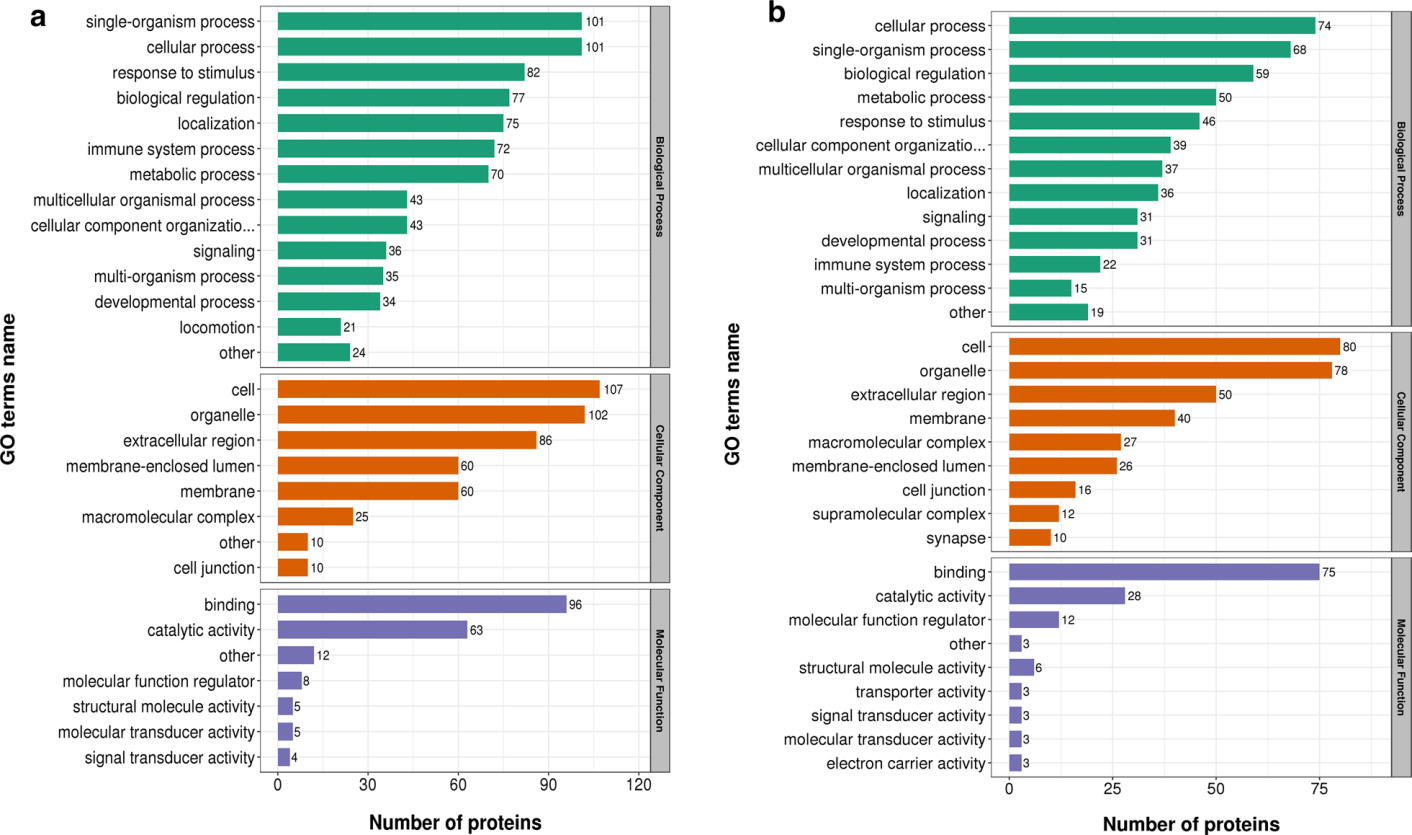
GO classifications of the Khib-modified proteins in the IgAN. **a** the GO classifications of upregulated proteins. **b** the GO classifications of downregulated proteins

To assess the 2-hydroxyisobutyrylated protein landscape in IgAN patients, the GO category (2nd Level) functional classification was assessed for all 2-hydroxyisobutyrylated proteins in three categories. Most of the upregulated 2-hydroxyisobutyrylated proteins were observed to be associated with the biological processes categories of single-organism process, cellular process, response to stimulus biological regulation, localization, and immune system process. Additionally, most of the DEPs were classified into the cell, organelle, and extracellular regions based on the cellular component category. In the molecular function classification, most of the DEPs were found to be involved in binding and catalytic activity (Fig. 2a). However, the majority of the downregulated proteins are involved in the single-organism process and cellular process categories; the cell, organelle, extracellular region, membrane and macromolecular complexes within the cellular component category; and binding, catalytic activity, and molecular function regulation within the molecular function classification (Fig. 2b). KOG category analysis was also performed through an NCBI database comparison. GO or KOG category functional classification revealed no significant difference in the DEPs between IgAN patients and NCs.

### GO, KEGG, and protein domain functional enrichment of the Khib proteome

**Fig. 3:**
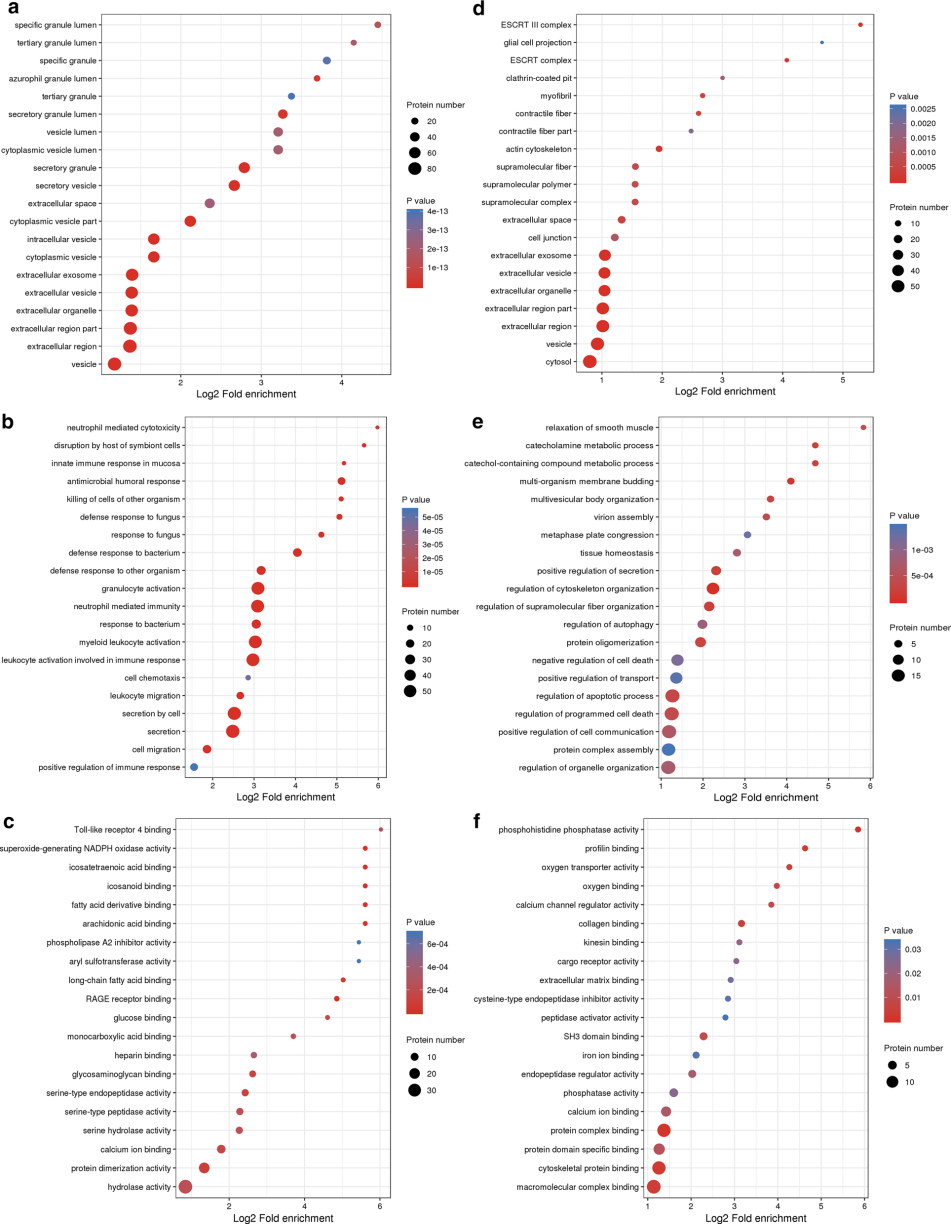
GO functional enrichment bubble plots of the differentially expressed proteins in the IgAN. The GO classifications of upregulated proteins in the cellular component **a**, the biological processes **b**, and the molecular functions. **c** The GO classifications of downregulated proteins in the cellular component **d**, the biological processes **e**, and the molecular functions (**f**)

We further performed a GO, KEGG pathway, and protein domain enrichment analysis with the intention of assessing whether there was a significant DEP enrichment trend in some functional categories. The upregulated DEPs were markedly enriched in the cellular component of secretory granule, secretory vesicle, cytoplasmic vesicle, and intracellular vesicle (Fig. 3a) and in the biological processes of secretion, secretion by cell, response to bacteria, and antimicrobial humoral response (Fig. 3b). Furthermore, they were identified as being enriched in the molecular functions of fatty acid derivative binding, eicosatetraenoic acid-binding, arachidonic acid-binding, and RAGE receptor binding (Fig. 3c). In contrast, there was no significant GO functional enrichment of any cellular component, biological process or molecular function for the downregulated DEPs (Fig. 3d–f).

**Fig. 4:**
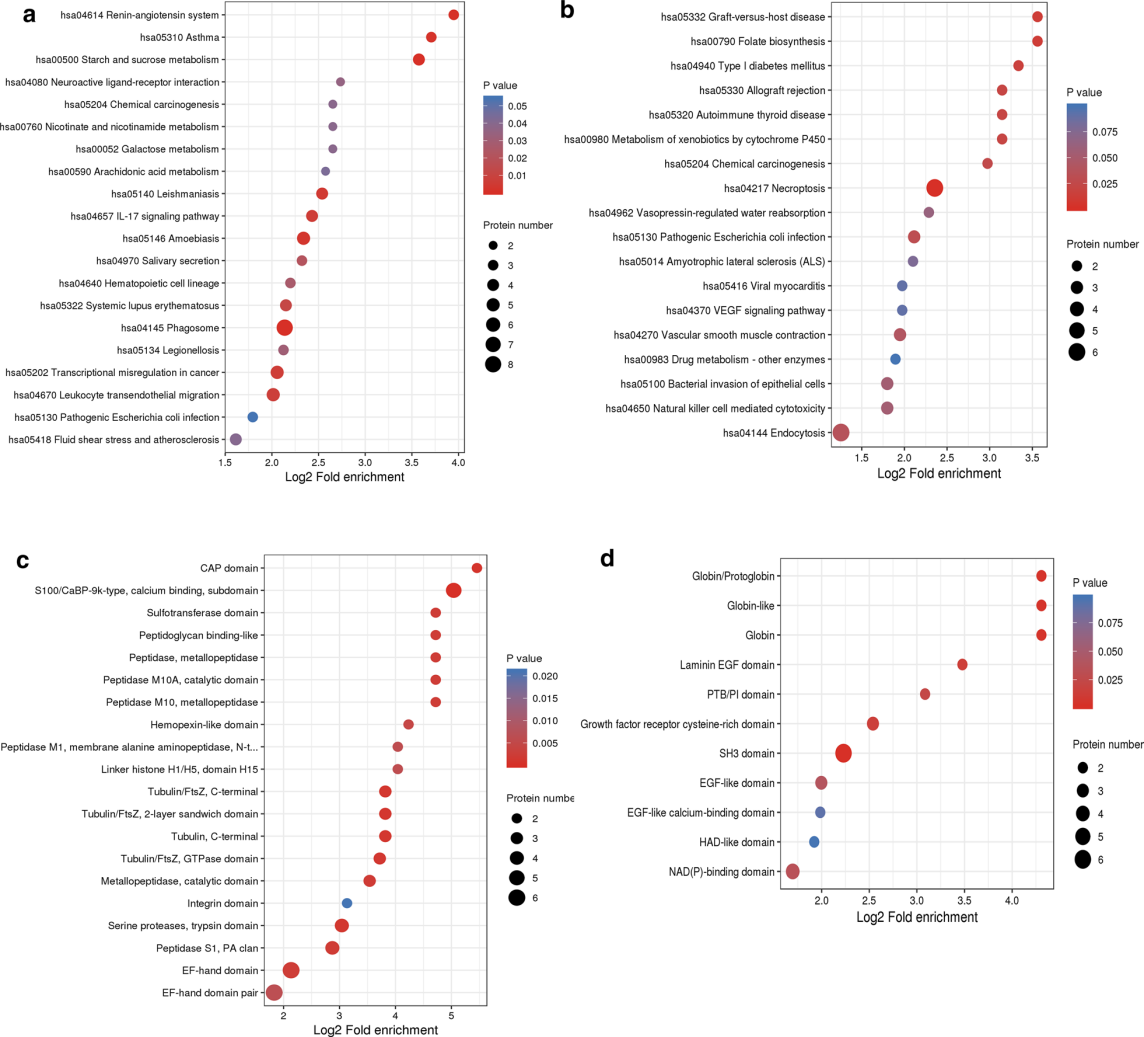
KEGG functional enrichment and protein domain bubble plots of the differentially expressed proteins in the IgAN. The upregulated proteins (**a**) and the downregulated proteins (**b**) in the KEGG functional enrichment. The upregulated proteins (**c**) and the downregulated proteins (**d**) in the protein domain

The KEGG-based functional enrichment analysis showed that the upregulated DEPs were significantly increased in 3 processes, including the hsa04614 renin-angiotensin system (Additional file 1: Fig. S1), hsa04145 phagosome, and hsa04657 IL-17 signaling pathway, which may have an important association with IgAN (Fig. 4a). There were other enriched KEGG functional pathways. For example, the upregulated DEPs in hsa05418 fluid shear stress and atherosclerosis may be associated with IgAN complications (such as the occurrence of cardiovascular disease), and those in hsa05134 legionellosis and hsa05140 leishmaniasis along with hsa05146 amoebiasis may be associated with the activation of IgAN pathophysiology. The downregulated DEPs were enriched in hsa04217 necroptosis (Fig. 4b).

Furthermore, the domains of the upregulated DEPs were only significantly enriched in the S100/CaBP − 9 k − type calcium-binding subdomain, as shown in Fig. 4c. The protein domains enriched in the downregulated DEPs included globin/protoglobin, globin-like, globin, and SH3 domain (Fig. 4d).

### Protein–protein interaction (PPI) network of the Khib proteins

**Fig. 5:**
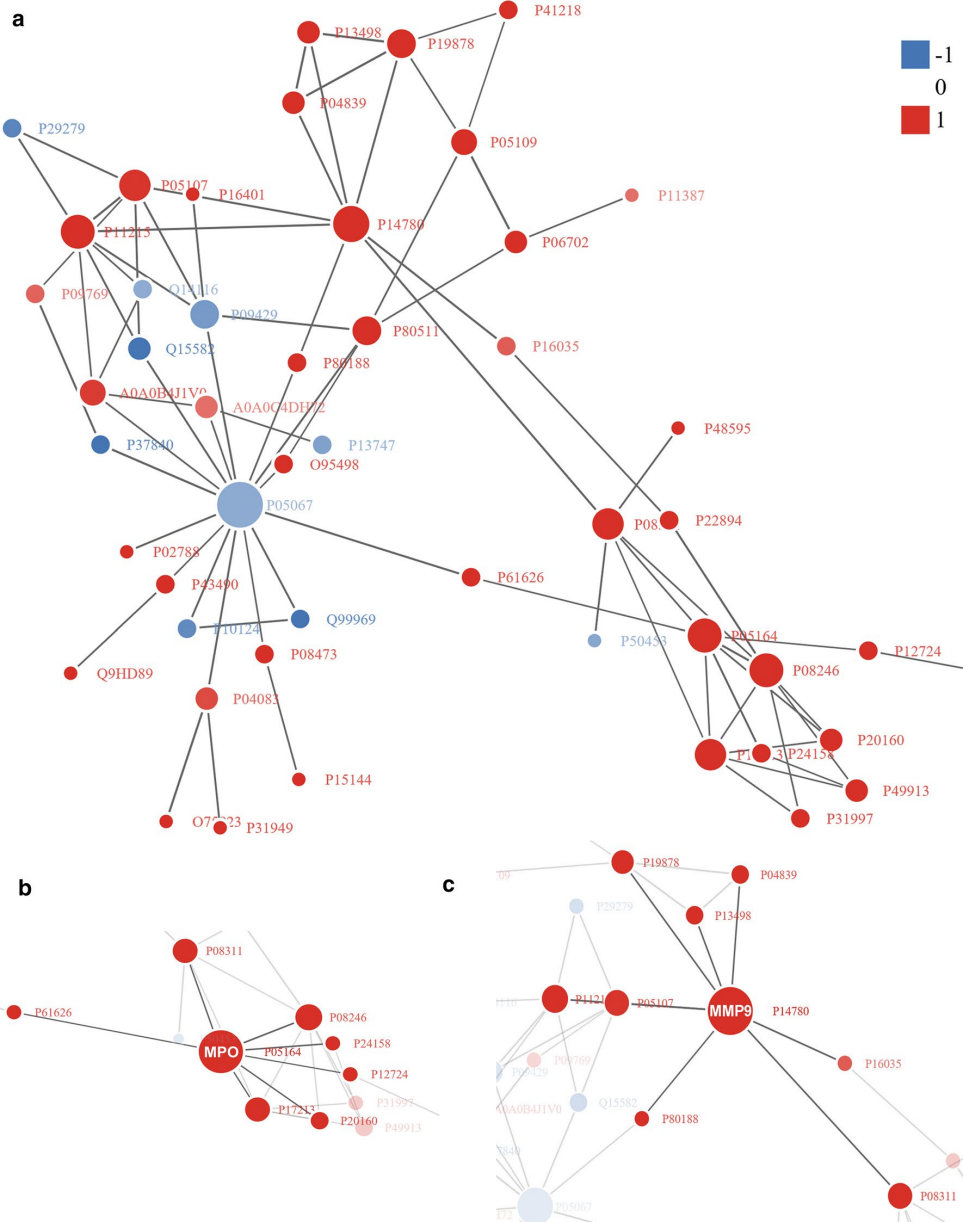
PPI network between the differentially expressed proteins in the IgAN. **a** Overview of the PPI network in the present study. **b** The PPI network of MMP9. **c** The PPI network of MPO

To broaden the landscape of the cellular processes regulated through 2-hydroxyisobutyrylation in IgAN patients, we constructed a PPI network model of the Khib proteins (Fig. 5a). DEPs were compared by the STRING protein–protein interaction database, and the top 50 tightly linked protein pairs were visually presented by the R package “networkD3” tool only when their confidence scores were greater than 0.7 (high confidence), identifying a varied cellular function landscape of 2-hydroxyisobutyrylated proteins in IgAN patients. Evidently, S100A8, MPO, MMP9, ITGAM, ITGB2 and BPI had the most interactions with other proteins (Fig. 5b). For instance, we found direct relationships between P14780 (MMP9) and other DEPs, along with P05164 (MPO; Fig. 5c). It seems that the physiological links among these 2-hydroxyisobutyrylated protein complexes contribute to their cooperation and coordination in IgAN patients.

### PRM-based validation

Eight proteins were quantified and confirmed by PRM analysis based on the results of TMT labeling quantitative analysis to verify the results of the whole-cell proteomics (Table 2). These proteins were selected according to their functional significance in the Khib proteome analysis in this study, and the ratio of protein abundance varied within a range. The identified proteins were quantified by PRM if at least 3 unique peptides were identified in each alternative protein. The PRM and TMT labeling quantification results were generally consistent, especially for the three following proteins: MPO (P05164), S100A9 (P06702) and S100A8 (P05109).

**Table 2:** The comparison of the quantification results between the TMT label and PRM methods

Protein Accession	Protein Name	IgA Raletive Abundance	NC Raletive Abundance	IgA/NC Ratio(PRM)	IgA/NC Ratio(TMT)
P05164	MPO	1.48	0.52	2.82	5.68
P06702	S100A9	1.71	0.29	5.80	5.66
P05109	S100A8	1.63	0.37	4.36	5.08
P62753	RPS6	0.97	1.03	0.94	1.54
P63104	YWHAZ	0.72	1.28	0.56	0.74
P05106	ITGB3	0.68	1.32	0.52	1.02
P14625	HSP90B1	0.56	1.44	0.39	0.83
P05556	ITGB1	0.47	1.53	0.31	1.07

### Proteome-wide analysis of Khib in PBMCs between IgAN patients and NCs

**Fig. 6:**
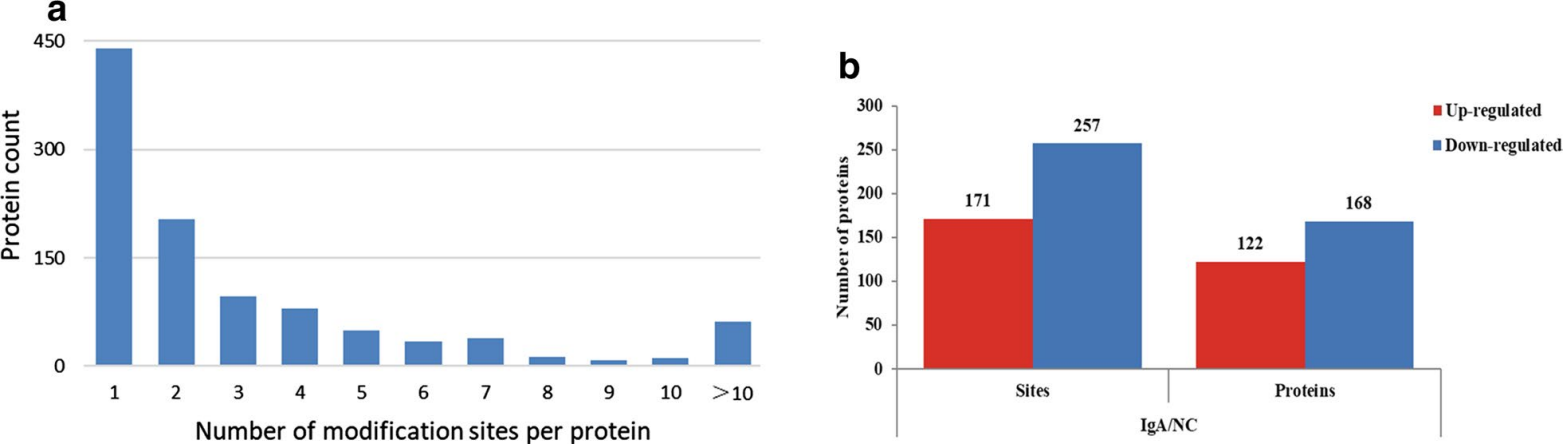
**a** Distribution of Khib in one protein. **b** Histogram of the number distribution of differentially expressed proteins and modification sites

In this study, we performed a Khib analysis in PBMCs from IgAN patients and NCs, which has not been reported previously. To ensure the high confidence of the results, we filtered the identification data using the criteria of localization probability > 0.75, FDR < 0.01 and score > 40. The effect of protein expression on the modified signal was removed by normalization to that of the protein quantitation group, and the normalized data were used for the subsequent bioinformatics analysis. In total, 3684 Khib sites distributed across 1036 proteins were identified, and 3132 sites on 894 proteins were quantifiable. The majority of the 2-hydroxyisobutyrylated proteins carried 1 to 4 Khib sites. Approximately 6% of the proteins carried more than 10 Khib sites (Fig. 6a), of which 94 Khib-modified sites were present in MYH9, being the greatest number of identified Khib sites on a single 2-hydroxyisobutyrylated protein.

### Identification of whole-cell Khib-modified proteins

In total, 122 Khib-modified proteins containing 171 sites with increased Khib modification and 168 Khib-modified proteins containing 257 specific sites were detected in the Khib DESs in IgAN patients (Fig. 6b). Among these DESs, we found that MYH9 mentioned above had 12 DESs, all of which had reduced Khib modification.

### GO functional classification of Khib -modified proteins and their subcellular localization

In the subcellular localization of Khib-modified proteins in this study, most of the upregulated Khib-modified proteins were distributed in the cytoplasm (49%), nucleus (25%), and mitochondria (7%). In addition, the majority of the downregulated Khib-modified proteins were located in the cytoplasm (42%) and nucleus (26%). According to the data, no significant differences were observed in the subcellular localization of the DEPs.

To acquire the Khib-modified proteome landscape in IgAN patients, GO functional classification was performed for Khib-modified proteins depending on the associated biological processes, molecular functions, and cellular components. Most of the upregulated Khib-modified proteins were associated with cellular processes, biological regulation, single-organism process, and metabolic process within the biological processes category. However, most of the downregulated proteins were associated with cellular processes and single-organism processes. Within the cellular component classification, the upregulated 2-hydroxyisobutyrylated proteins, for the most part, were associated with cell, organelle, macromolecular complex, extracellular region, membrane and membrane-enclosed lumen. In addition, most of the downregulated proteins were associated with cell, organelle, membrane, extracellular region, macromolecular complex and membrane-enclosed lumen. Moreover, within the molecular function category, most of the upregulated DEPs were associated with binding, catalytic activity, structural molecule activity, and molecular function regulator. In contrast, most of the downregulated DEPs were associated with binding, catalytic activity, structural molecule activity, and molecular function regulator. There were no significant differences in the GO functional classification of the DEPs, implying that Khib modification may have wide-ranging biological functions.

### GO, KEGG, and protein domain functional enrichment of the Khib-modified proteins

**Fig. 7:**
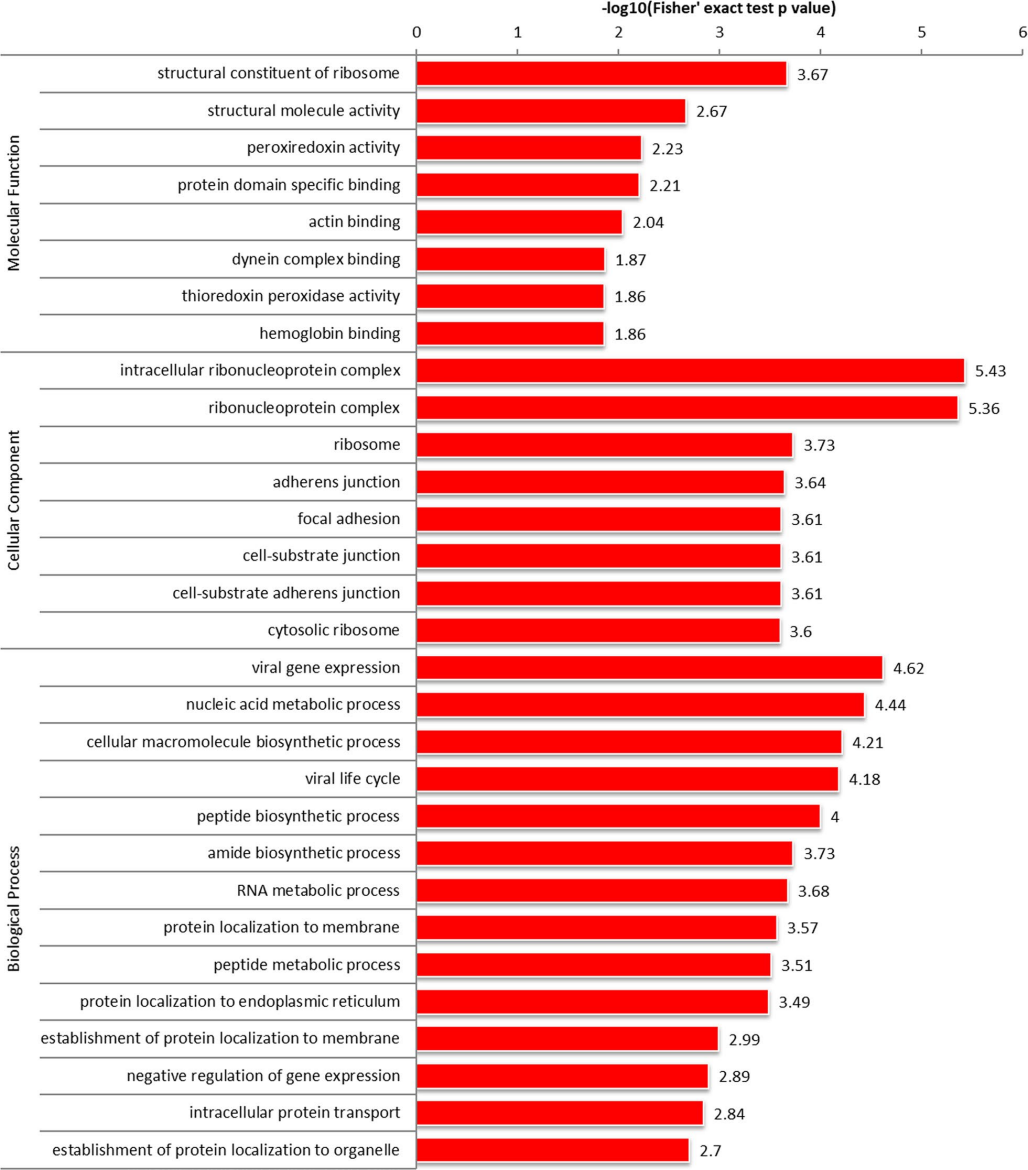
GO-based functional enrichment analysis of upregulated Khib proteins in terms of cellular component, molecular function, and biological process

The upregulated 2-hydroxyisobutyrylated proteins were predominantly clustered in the cellular component of intracellular ribonucleoprotein complex, and ribonucleoprotein complex; molecular functions including structural constituent of ribosomes, structural molecule activity, peroxiredoxin activity and actin-binding; and biological processes including viral gene expression, nucleic acid metabolic process, cellular macromolecule biosynthetic process, viral life cycle and peptide biosynthetic process (Fig. 7). The downregulated 2-hydroxyisobutyrylated proteins were obviously clustered in the cellular component of cell membrane projection, membrane ruffles, leading edge membrane; molecular functions including cytoskeletal protein binding, actin binding and cell adhesion molecule binding; and biological processes including nuclear division, homotypic cell–cell adhesion and mitotic nuclear division.

**Fig. 8:**
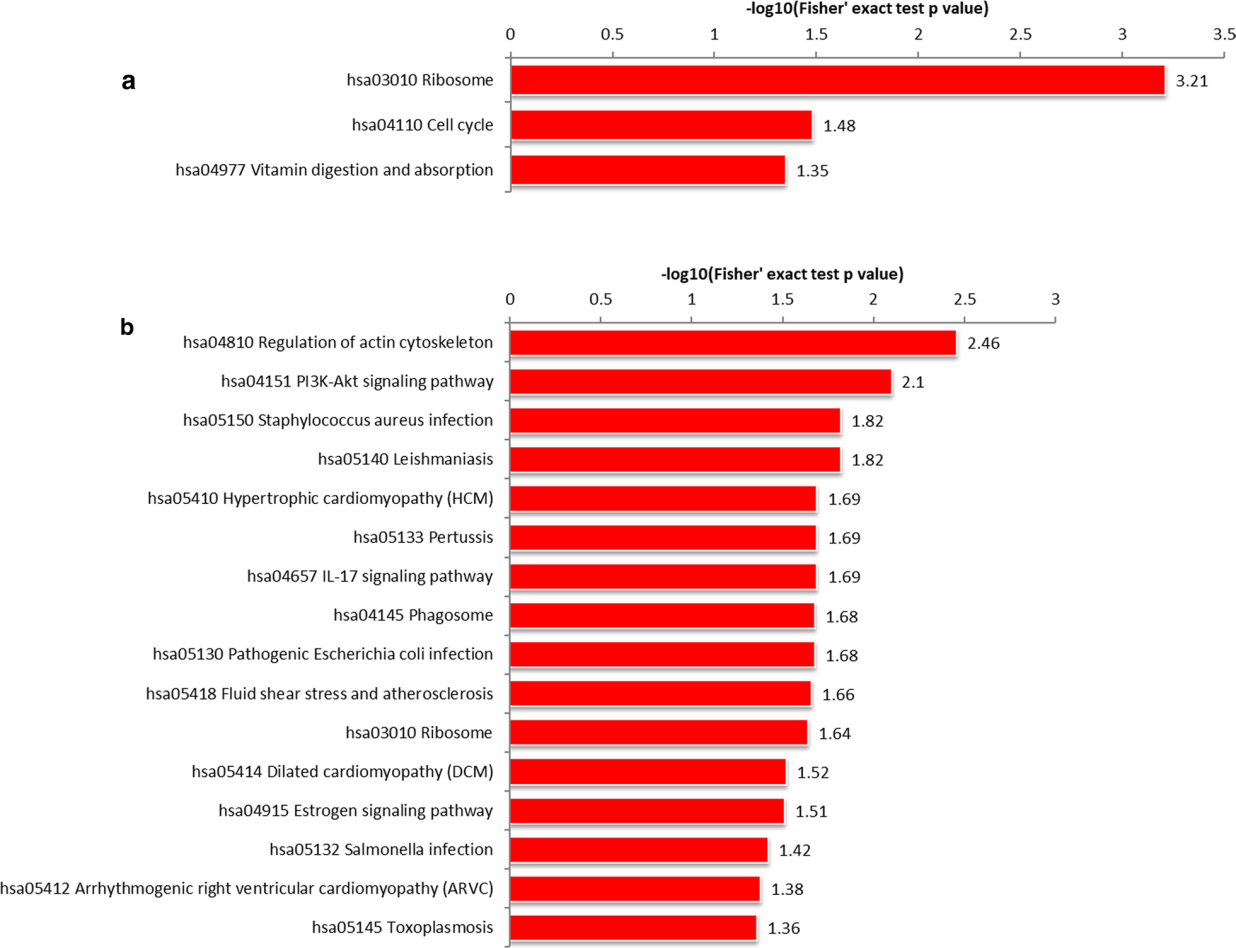
**a** KEGG-based functional enrichment analysis of upregulated Khib proteins. **b** KEGG-based functional enrichment analysis of downregulated Khib proteins

KEGG-based functional enrichment analysis illustrated that the upregulated 2-hydroxyisobutyrylated proteins were enriched in processes (as shown in Fig. 8a) including ribosomes and the cell cycle. The downregulated 2-hydroxyisobutyrylated proteins were enriched in 16 processes, including regulation of the actin cytoskeleton, IL-17 signaling pathway, PI3K-Akt signaling pathway, fluid shear stress and atherosclerosis, and ribosome and phagosome, which have important associations with IgAN complications (Fig. 8b).

Furthermore, upregulated 2-hydroxyisobutyrylated proteins were enriched in domains such as nucleic acid-binding, OB-fold; nucleotide-binding alpha–beta plait domain; K homology domain, type 1; K homology domain; translation protein SH3-like domain; FERM, N-terminal; zinc finger, PHD-type; zinc finger, PHD-finger; rubredoxin-type fold; heat shock chaperonin-binding and FERM domain, demonstrating the competing and unique roles of Khib-modified proteins. Downregulated 2-hydroxyisobutyrylated proteins were observed to be enriched in protein domains including histidine kinase-like ATPase, C-terminal domain, integrin domain, heat shock protein Hsp90, N-terminal, calponin homology domain, ribosomal protein S5 domain 2-type fold; S100/CaBP-9 k-type, calcium-binding, subdomain, EF-hand domain pair and FERM, N-terminal.

### GO, KEGG, and protein domain cluster analyses

Based on the analyses of DEPs in the different comparison groups for the GO classification, KEGG pathway and protein domain enrichment, we applied cluster analyses to research the correlation between the function of DEPs in the comparison groups.

For the GO analysis cellular component, there was no enrichment in upregulated 2-hydroxyisobutyrylated proteins, whereas the downregulated 2-hydroxyisobutyrylated proteins were mainly enriched in neuromuscular junction, cell projection, cytoskeletal part, cytoskeleton, plasma membrane part, plasma membrane region, filopodium and membrane ruffles (Additional file 2: Fig. S2 and Additional file 3: Fig. S3).

The biological process enrichment of 2-hydroxyisobutyrylation was carried out and upregulated 2-hydroxyisobutyrylated proteins tended to be enriched in cellular functions such as biosynthetic process. We found that the downregulated proteins were mainly associated with homotypic cell–cell adhesion, animal organ development, response to fungus, integrin-mediated signaling pathway, regulation of plasma membrane organization and spindle organization (Additional file 4: Fig. S4 and Additional file 5: Fig. S5).

Moreover, the upregulated proteins were mainly clustered in the structural constituent of ribosomes, structural molecule activity, peroxiredoxin activity and various protein binding processes. Within the category of molecular function, the downregulated 2-hydroxyisobutyrylated proteins were specifically involved in the cellular binding process (Additional file 6: Fig. S6 and Additional file 7: Fig. S7).

The KEGG enrichment analysis of all DEPs identified the PI3K-Akt signaling pathway. Accordingly, the upregulated 2-hydroxyisobutyrylated proteins present in the most prominent enriched pathways were associated with the ribosome, cell cycle, and vitamin digestion and absorption, whereas the downregulated proteins were enriched in the ribosome and phagosome; diseases such as *Staphylococcus aureus* infection, pathogenic *Escherichia coli* infection, hypertrophic cardiomyopathy (HCM) and fluid shear stress and atherosclerosis; cellular signaling pathways such as the IL-17 signaling pathway and the PI3K-Akt signaling pathway, and cellular metabolism (Additional file 8: Fig. S8 and Additional file 9: Fig. S9).

The protein domains of the 2-hydroxyisobutyrylated proteins were evaluated. There were extremely upregulated enriched genes containing heat shock chaperonin-binding, rubredoxin-type fold, nucleic acid-binding, OB-fold and K homology domains. The highly downregulated proteins were enriched in the integrin domain; S100/CaBP-9 k-type, calcium-binding, subdomain; ribosomal protein S5 domain 2-type fold; histidine kinase-like ATPase, C-terminal domain and heat shock protein Hsp90, N-terminal domain, similar to the results from functional classification of the protein domains (Additional file 10: Fig. S10 and Additional file 11: Fig. S11).

## Discussion

Mesangial IgA deposition is observable in the kidney biopsies of IgAN patients. IgA is a prevalent antibody type in humans and protects against pathogens [22]. IgA1 is one of the subclasses of IgA and only IgA1 is observed in the mesangial deposits in the kidney biopsies of IgAN patients [23]. One kind of human IgA1 is the monomeric form, which occurs mostly in the circulation. According to a current widely-accepted definition of the pathophysiology of IgAN development, the appearance of immune complexes and consequent mesangial deposition are necessary to initiate glomerular injury. These immune complexes contain circulating Gd-IgA1, which has aberrant protein glycosylation, is a critical effector molecule for IgAN and might be a favorable biomarker for differentiating IgAN from other glomerular diseases [24–28]. Glycosylation is a common type of PTM that is involved in many biological processes, such as protein–protein interactions [29, 30]. Khib is another PTM and its biochemical function in IgAN patients is poorly understood. To the best of our knowledge, this is the first study to demonstrate Khib modifications in IgAN patients. This study expands the current understanding of the role of lysine 2-hydroxyisobutyrylation in the pathophysiology of IgAN.

**Fig. 9:**
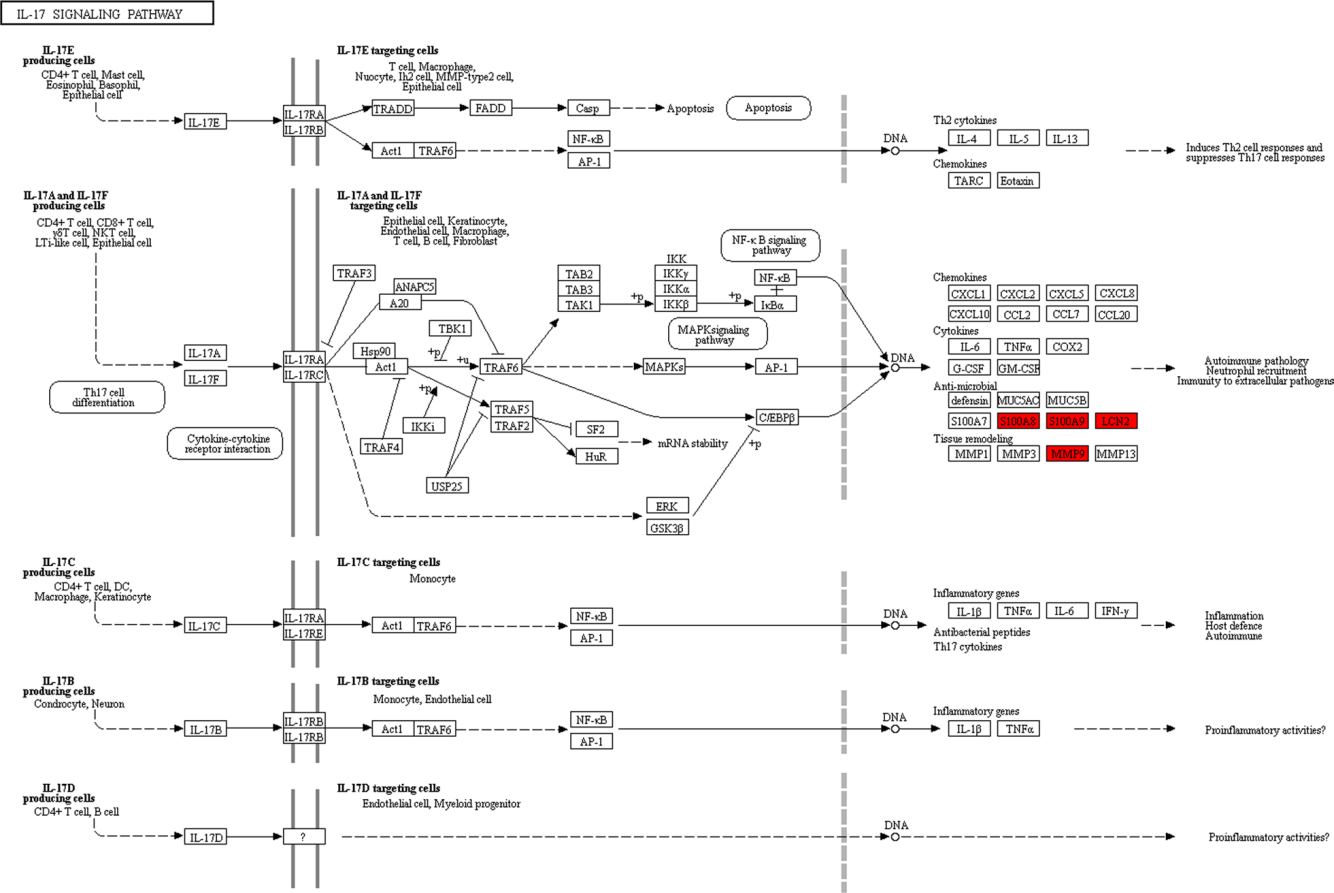
The IL-17 signaling pathway cascades obtained from the global proteome data by KEGG pathway analysis. The proteins in red are upregulated

In this study, we observed that most of the DEPs were increased in IgAN patients. The abundance of LCN2, MMP9, S100A9 and S100A8 was significantly increased in PBMCs and LCN2, MMP9, S100A9 and S100A8 occurred in the IL-17 signaling pathway in the KEGG pathway analysis (Fig. 9); this pathway has various functions in autoimmune pathology, neutrophil recruitment and immunity to extracellular pathogens. The IL-17 signaling pathway is considered to be highly upregulated in the inflammatory tissues of patients with autoimmune diseases [31–33], and IL-17-specific antibody treatments are remarkably effective for treating certain inflammatory diseases, such as Crohn's disease [34–36]. As outlined above, the upregulated DEPs and the IL-17 signaling pathway may also play a role in the pathology of IgAN. We also observed that the MAPK/ERK signaling pathway, which is downstream of the IL-17 pathway, was not activated. According to an evaluation of renal biopsy-proven IgAN patients, the MAPK/ERK signaling pathway is present in the mesangium of patients presenting with elevated blood pressure and over 1 g/day proteinuria but absent in biopsy specimens of patients with IgAN and modest proteinuria (< 1 g/day) [37]. We found that the Khib modification of the DESs in S100A9 and S100A8 of the IL-17 signaling pathway was significantly reduced, especially K38 of S100A9 (IgA/NC ratio: 0.435, P value < 0.001).

**Fig. 10:**
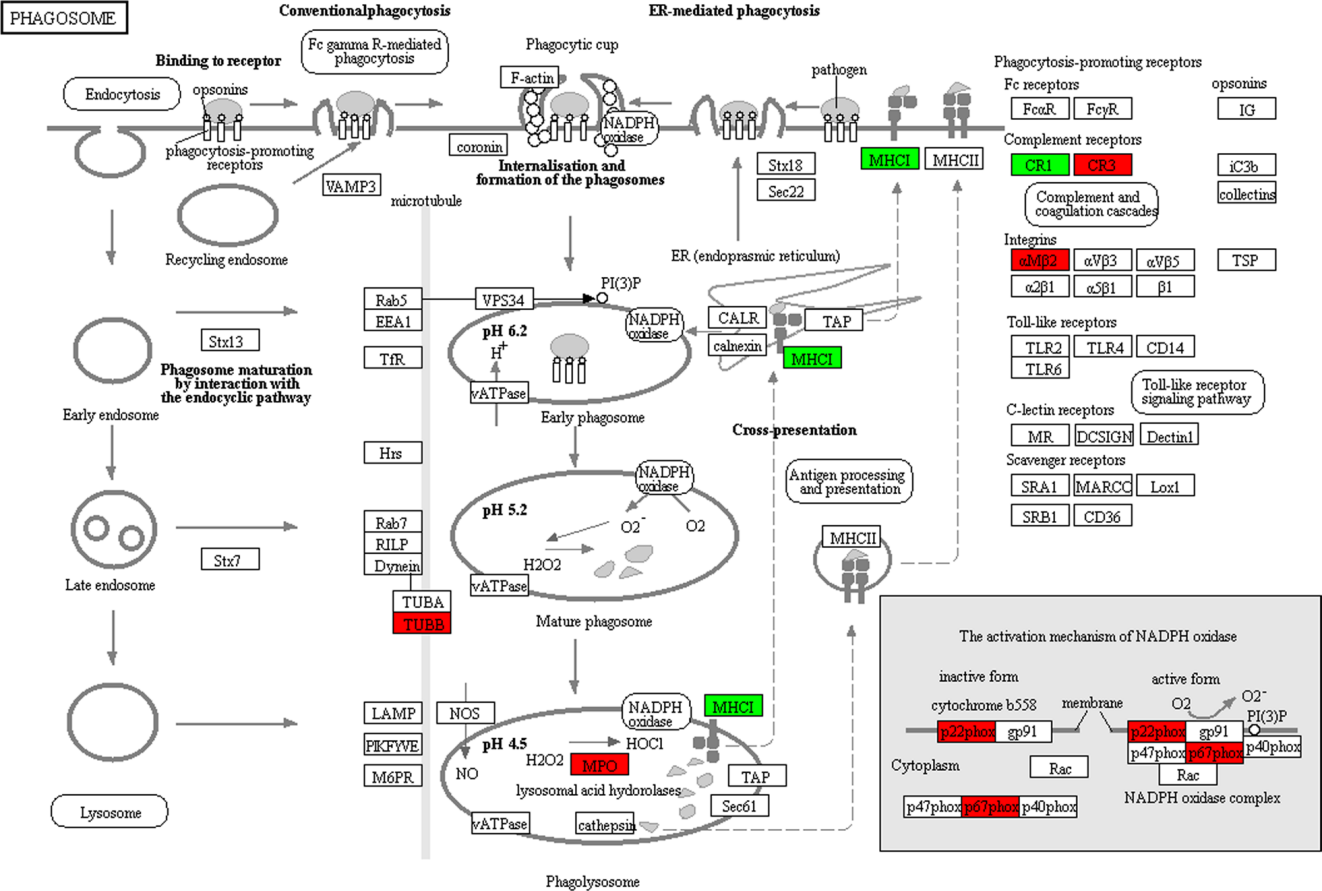
The phagosome pathway cascades obtained from the global proteome data by KEGG pathway analysis. The proteins in red are upregulated, and the proteins in green are downregulated

We found that MPO is one of the top tightly interacting proteins confirmed by the protein–protein interaction network and a significantly upregulated protein (IgA/NC ratio: 5.675); furthermore, MPO is associated with the phagosome pathway [38], which is one of the three processes enriched in the upregulated 2-hydroxyisobutyrylated proteins, as shown by the KEGG-based functional enrichment analysis (Fig. 10). The quantitative data demonstrated that the levels of MPO (P05164), CR3 (P05107 and P11215) and TUBB (P68371) from this pathway were specifically upregulated in PBMCs from IgAN patients compared to NCs. Phagocytosis is indispensable for the degradation and presentation of antigens, activation of the adaptive immune response and the elimination of pathogenic microorganisms. A convincing study revealed that autophagy is closely associated with the pathological form of nephritis [39]. Increasing numbers of studies have demonstrated that podocyte injury plays a significant role during the deterioration of IgAN. Moreover, further reports have shown that podocyte detachment from the glomerular basal membrane occurs in IgAN [40]. In a retrospective study, Liang et al. investigated the number of autophagosomes in podocytes from the kidney tissue of IgAN patients and found that it was lower in the nephropathy group compared to the control group [41], which is the opposite the results identified in PBMCs. This finding has obscured whether there is a connection or contrast between podocytes and PBMCs.

**Fig. 11:**
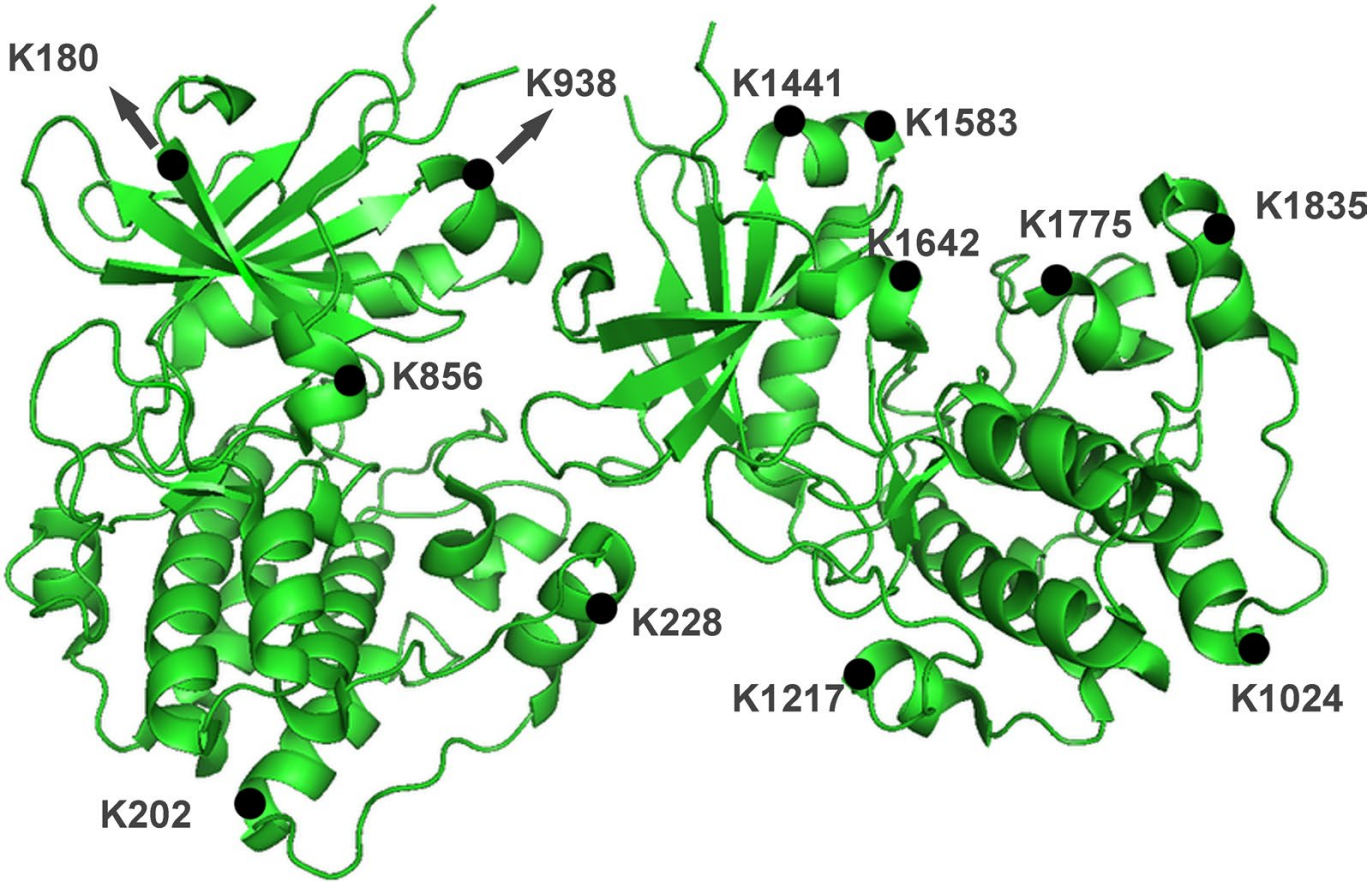
A sketch map for MYH9 with 12 downregulated DESs

Many of the identified Khib-modified DEPs have not previously been associated with IgAN. Another example is MYH9 (Fig. 11), which has 94 Khib-modified sites among which 12 downregulated DESs have been reported to be associated with vascular smooth muscle contraction and relaxation [42]. We look forward to witnessing what further research efforts on these abovementioned proteins can contribute to revealing and clarifying the mechanism underlying the pathogenesis of IgAN.

Despite the power of proteomics profiles and use of bioinformatics analyses, there are potential limitations to our work. We aimed to preliminarily understand the characteristics of serological PBMC in different stages of IgAN, and, as a result, patients with different stages of renal failure had been recruited. IgAN is a disease of complex etiology, and it is possible that the results in this study may be reflected by different stages of renal failure caused by IgAN.

## Conclusions

Our quantitative proteomics study presents a comprehensive landscape of the proteomic features of PBMCs from patients with IgAN. The proteins mentioned in the IL-17 signaling pathway and phagosomes may also be potential biomarkers for use in noninvasive diagnostic tests and are probably factors involved in the pathogenesis of IgAN. Based on previous studies, we demonstrate that the IL-17 signaling pathway and phagosomes in PBMCs might be involved in the pathogenesis of IgAN and may represent potential biomarkers for noninvasive diagnostic tests or even therapeutic targets. Functional clustering analysis also indicated that the immune effector cells in PBMCs from patients were activated. Many proteins identified in this study have never been associated with IgAN, and future studies are needed to analyze and verify these changes and determine their significance for the diagnosis and treatment of IgAN.

## Supplementary Information

**Additional file 1: Fig. S1** Protein domains analysis of the downregulated Khib-modified proteins in the IgAN.

**Additional file 2: Fig. S2** The renin-angiotensin system pathway cascades obtained from the global proteome data by KEGG pathway analysis. The proteins in red are upregulated.

**Additional file 3: Fig. S3** Functional enrichment analysis of the upregulated Khib-modified proteins in the IgAN based on the GO analysis cellular component.

**Additional file 4: Fig. S4** Functional enrichment analysis of the downregulated Khib-modified proteins in the IgAN based on the GO analysis cellular component.

**Additional file 5: Fig. S5** Functional enrichment analysis of the upregulated Khib-modified proteins in the IgAN based on the GO analysis biological process.

**Additional file 6: Fig. S6** Functional enrichment analysis of the downregulated Khib-modified proteins in the IgAN based on the GO analysis biological process.

**Additional file 7: Fig. S7** Functional enrichment analysis of the upregulated Khib-modified proteins in the IgAN based on the GO analysis molecular function.

**Additional file 8: Fig. S8** Functional enrichment analysis of the downregulated Khib-modified proteins in the IgAN based on the GO analysis molecular function.

**Additional file 9: Fig. S9** KEGG enrichment analysis of the upregulated Khib-modified proteins in the IgAN.

**Additional file 10: Fig. S10** KEGG enrichment analysis of the downregulated Khib-modified proteins in the IgAN.

**Additional file 11: Fig. S11** Protein domains analysis of the upregulated Khib-modified proteins in the IgAN.

## Data Availability

Please contact author for data requests.

## Abbreviations

Khib: Lysine 2-hydroxyisobutyrylation
IgAN: Immunoglobulin A nephropathy
PTMs: Posttranslational modification
PBMCs: Peripheral blood mononuclear cells
Gd-IgA1: Galactose-deficient O-glycan iga1
NC: Normal control
FDR: False-positive discovery rate
GO: Gene ontology
KEGG: Kyoto encyclopedia of genes and genomes
PPI: Protein–protein interaction
DEPs: Differentially expressed proteins
DESs: Differentially expressed sites
