# Supplementary figures and images for: Quantitative proteomics analysis of lysine 2-hydroxyisobutyrylation in IgA nephropathy
Source: Clin Proteomics. 2021 Feb 8;18:7. doi: 10.1186/s12014-021-09314-0 (PMC7869230; doi:10.1186/s12014-021-09314-0)

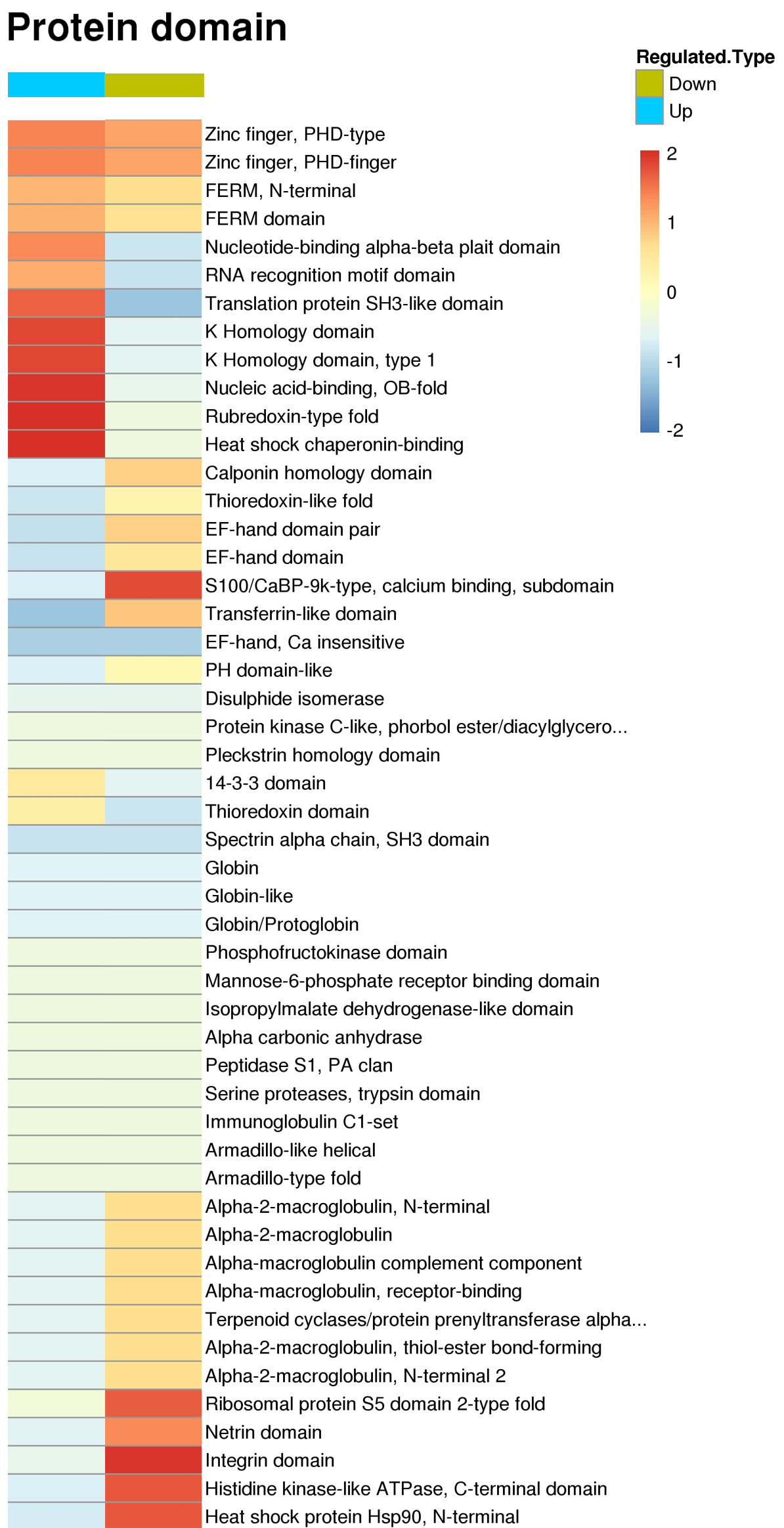

Supplement: Supplementary file 1 — Additional file 1: Fig. S1 Protein domains analysis of the downregulated Khib-modified proteins in the IgAN. [file 12014_2021_9314_MOESM1_ESM.tif]

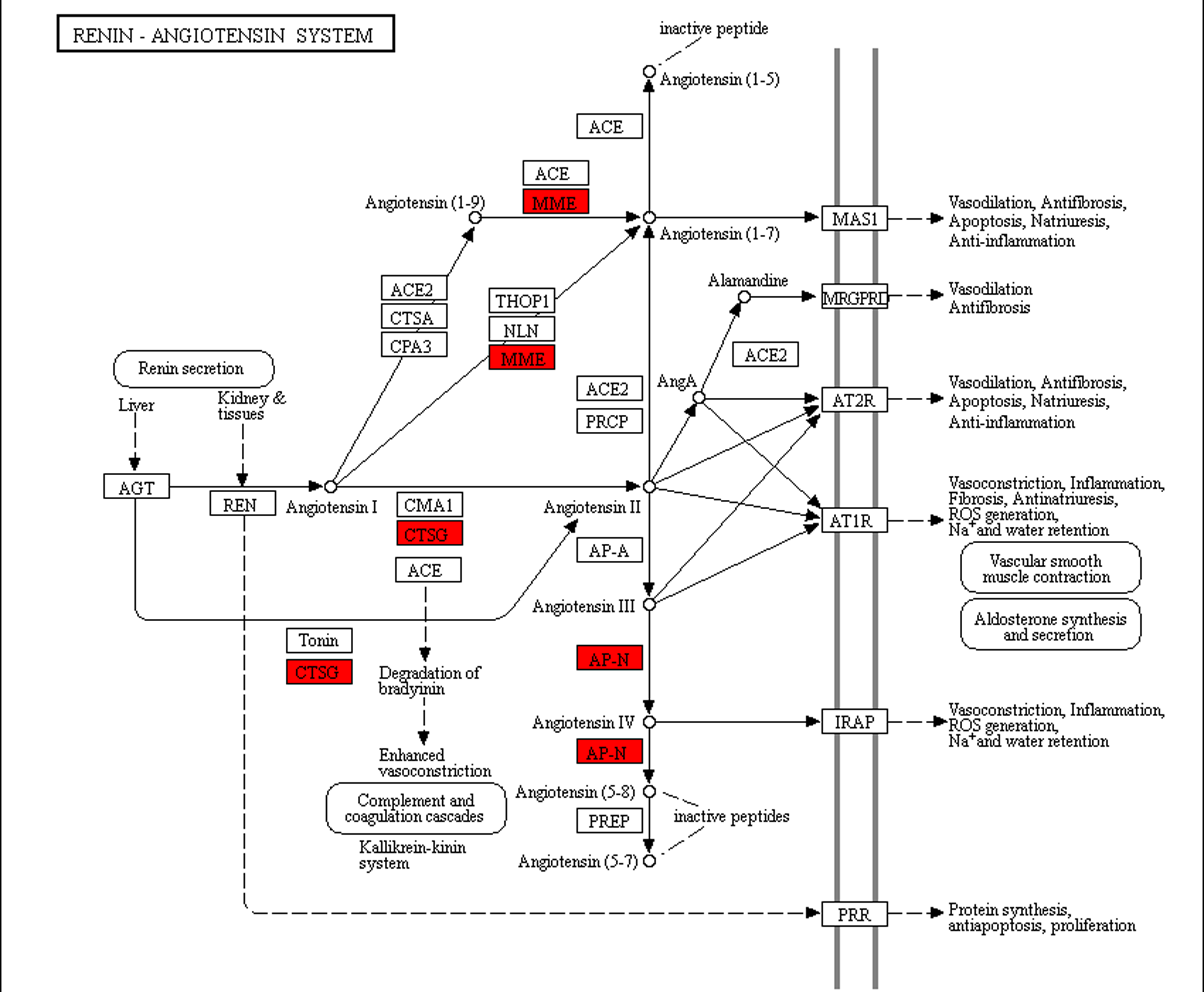

Supplement: Supplementary file 2 — Additional file 2: Fig. S2 The renin-angiotensin system pathway cascades obtained from the global proteome data by KEGG pathway analysis. The proteins in red are upregulated. [file 12014_2021_9314_MOESM2_ESM.tif]

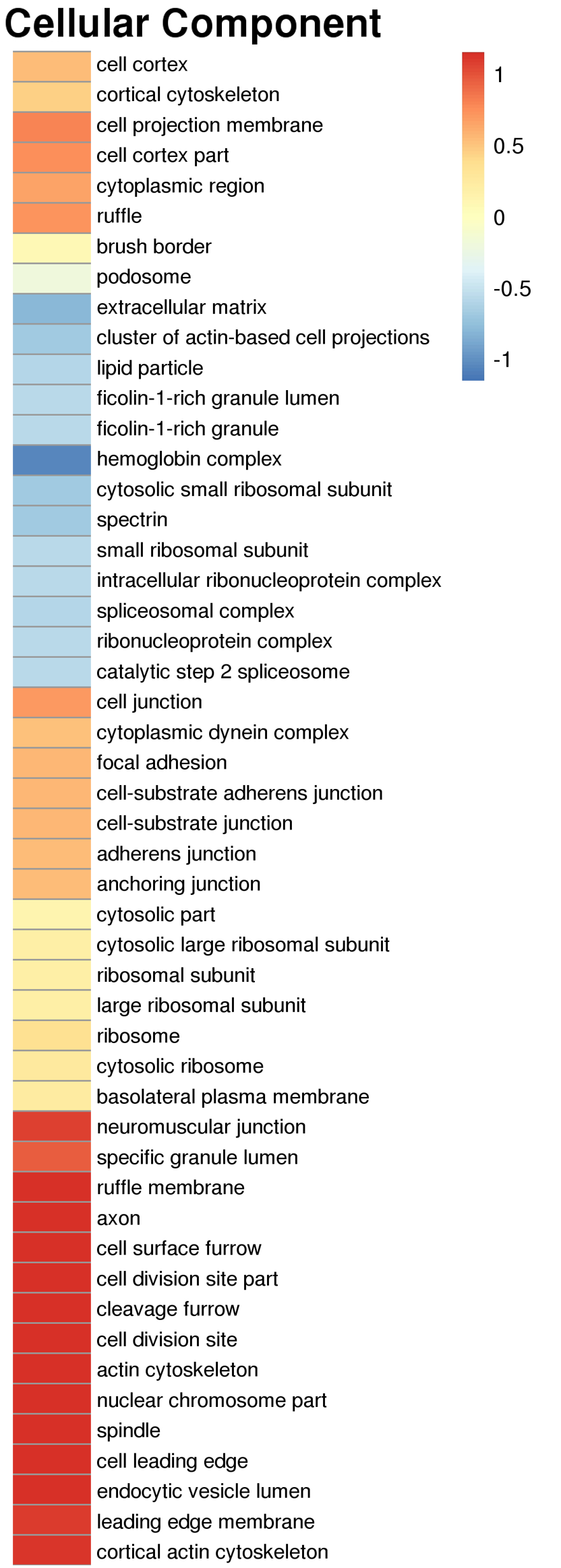

Supplement: Supplementary file 3 — Additional file 3: Fig. S3 Functional enrichment analysis of the upregulated Khib-modified proteins in the IgAN based on the GO analysis cellular component. [file 12014_2021_9314_MOESM3_ESM.tif]

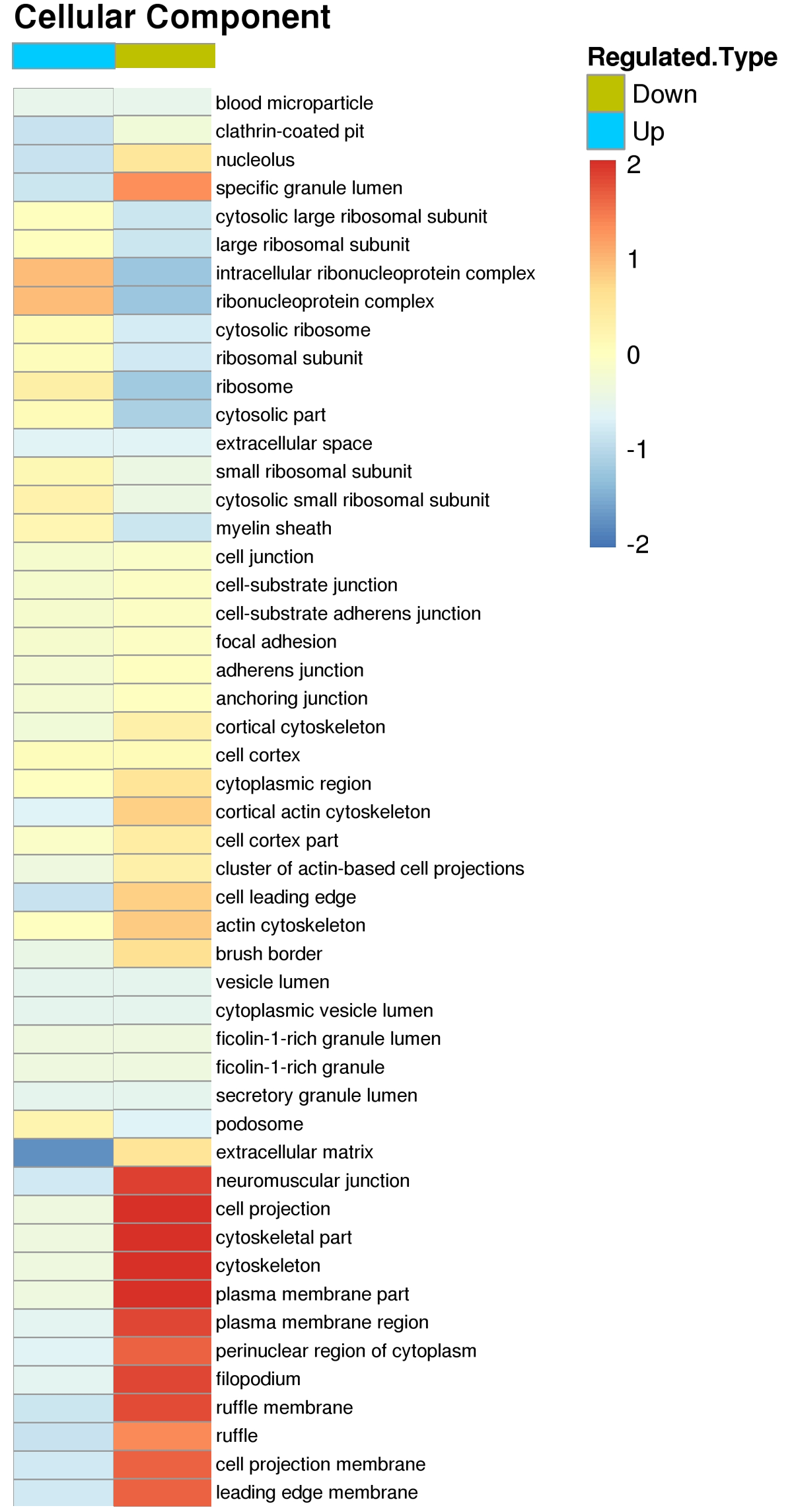

Supplement: Supplementary file 4 — Additional file 4: Fig. S4 Functional enrichment analysis of the downregulated Khib-modified proteins in the IgAN based on the GO analysis cellular component. [file 12014_2021_9314_MOESM4_ESM.tif]

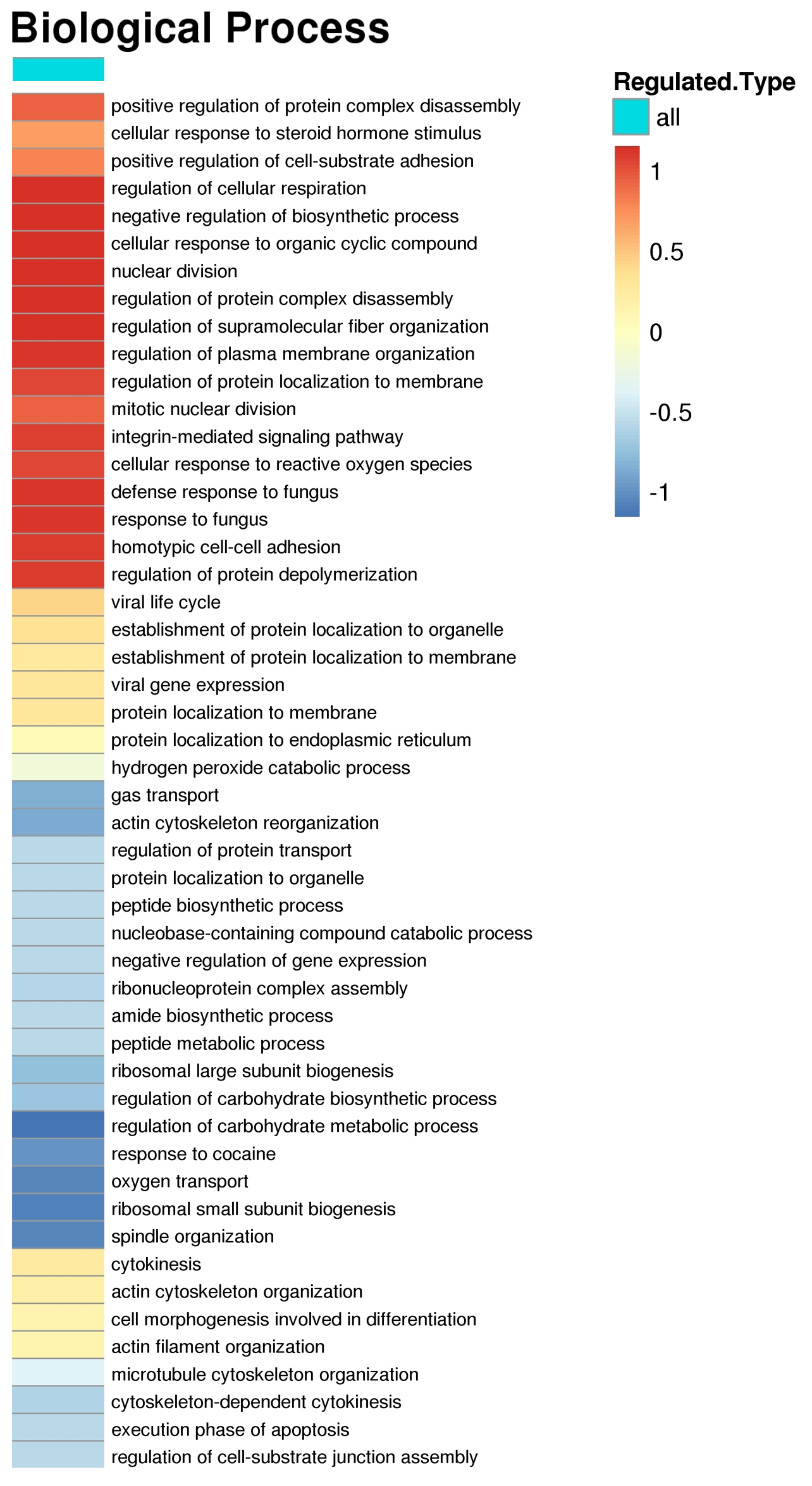

Supplement: Supplementary file 5 — Additional file 5: Fig. S5 Functional enrichment analysis of the upregulated Khib-modified proteins in the IgAN based on the GO analysis biological process. [file 12014_2021_9314_MOESM5_ESM.tif]

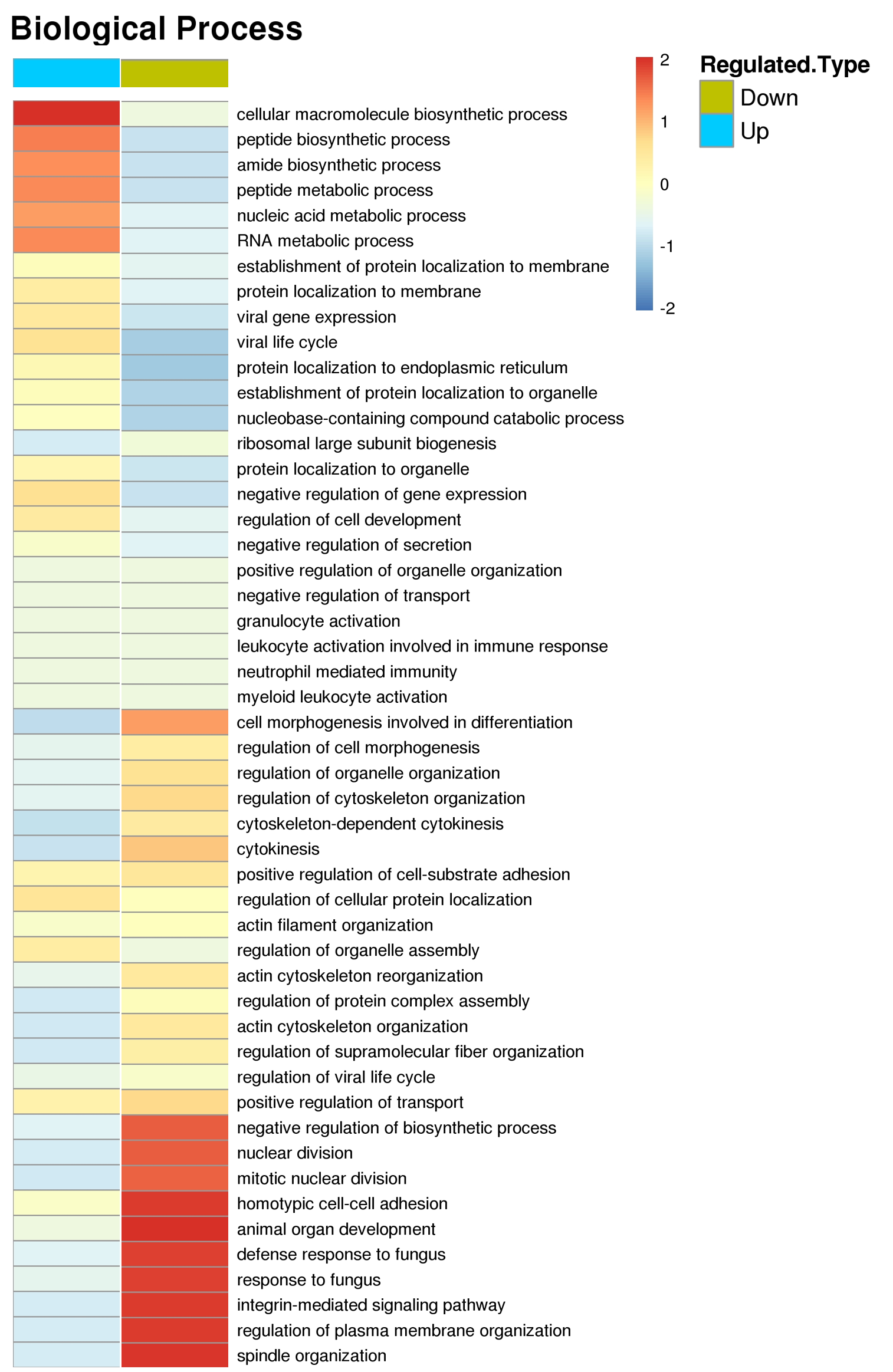

Supplement: Supplementary file 6 — Additional file 6: Fig. S6 Functional enrichment analysis of the downregulated Khib-modified proteins in the IgAN based on the GO analysis biological process. [file 12014_2021_9314_MOESM6_ESM.tif]

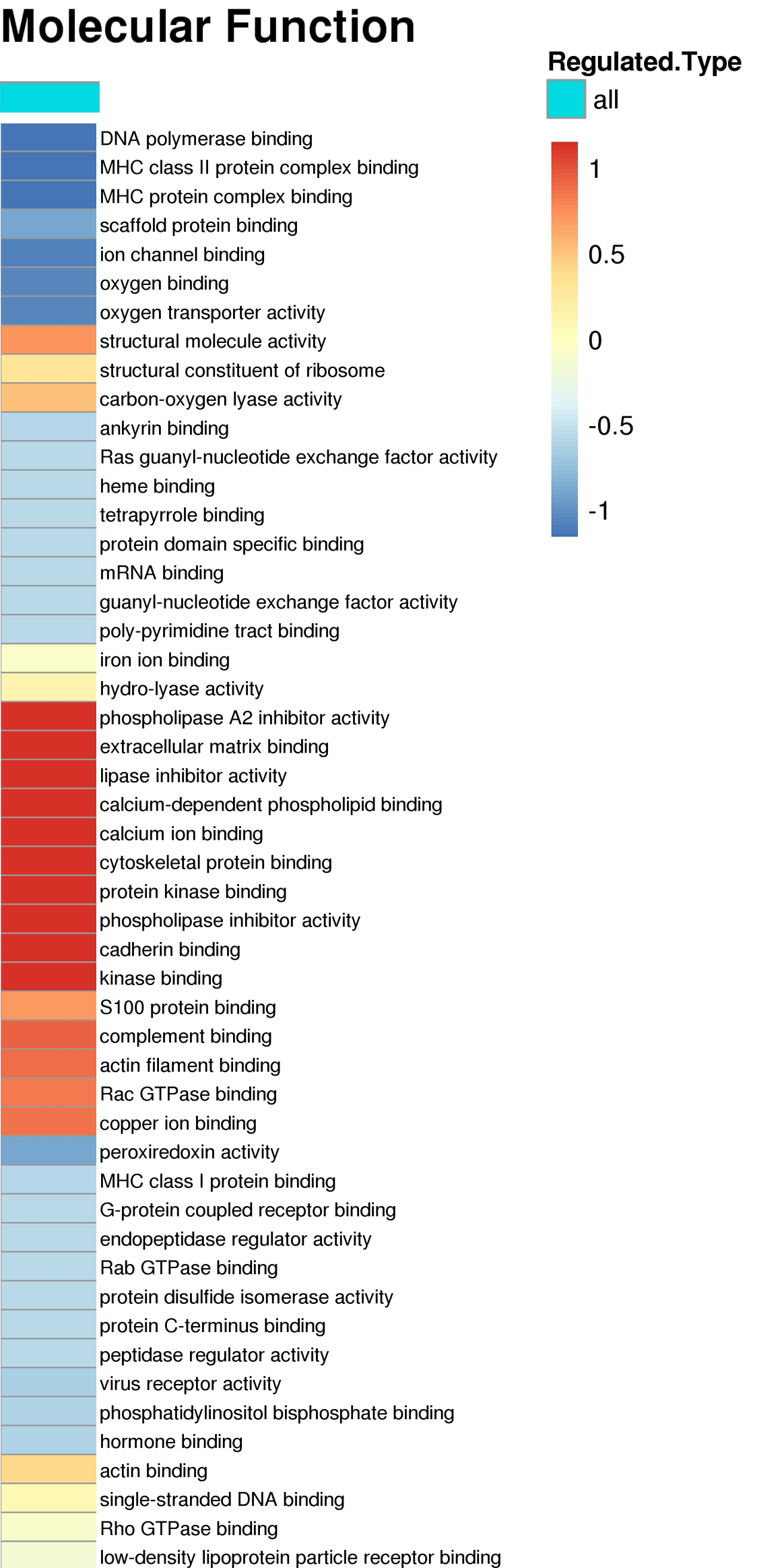

Supplement: Supplementary file 7 — Additional file 7: Fig. S7 Functional enrichment analysis of the upregulated Khib-modified proteins in the IgAN based on the GO analysis molecular function. [file 12014_2021_9314_MOESM7_ESM.tif]

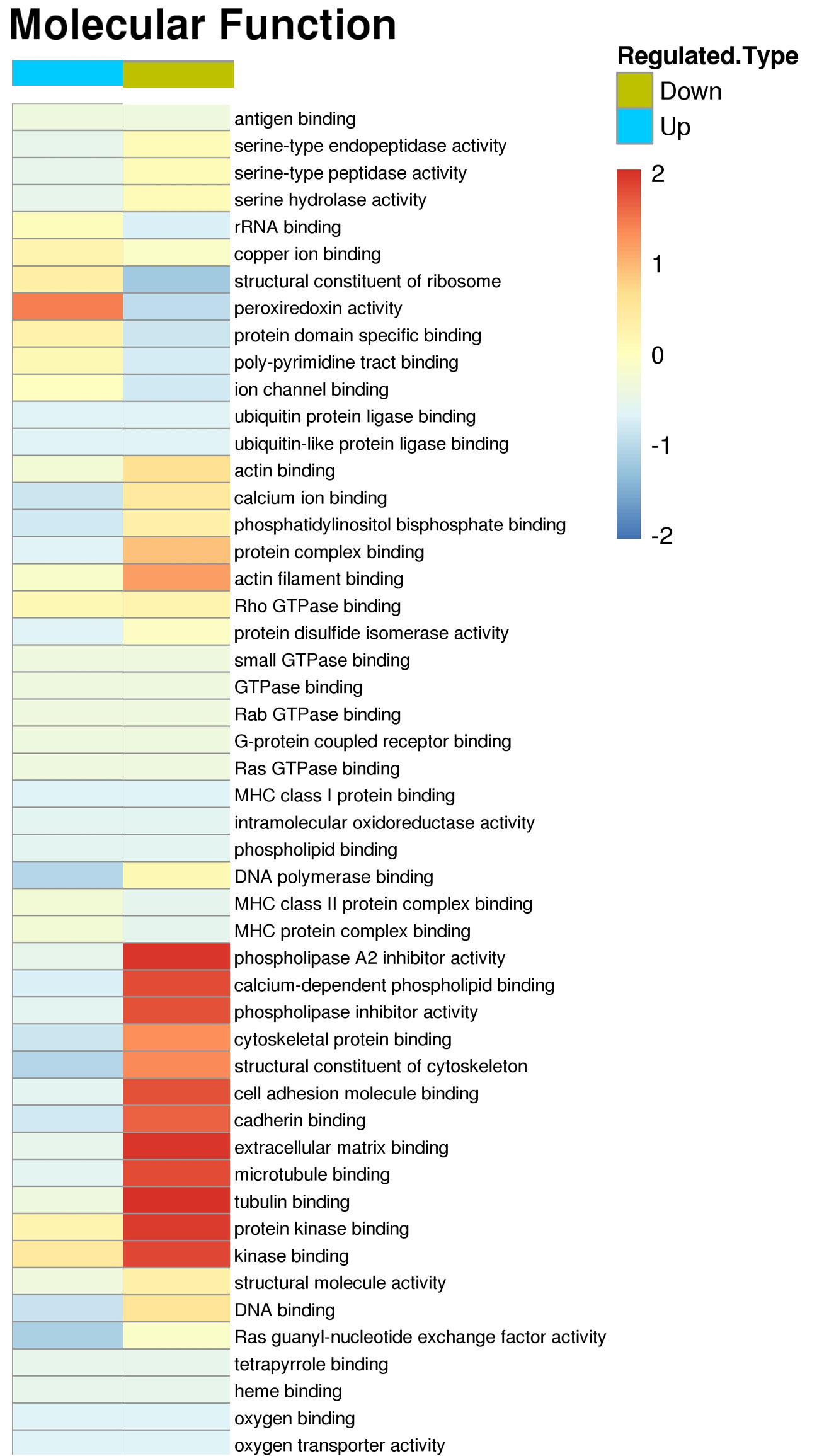

Supplement: Supplementary file 8 — Additional file 8: Fig. S8 Functional enrichment analysis of the downregulated Khib-modified proteins in the IgAN based on the GO analysis molecular function. [file 12014_2021_9314_MOESM8_ESM.tif]

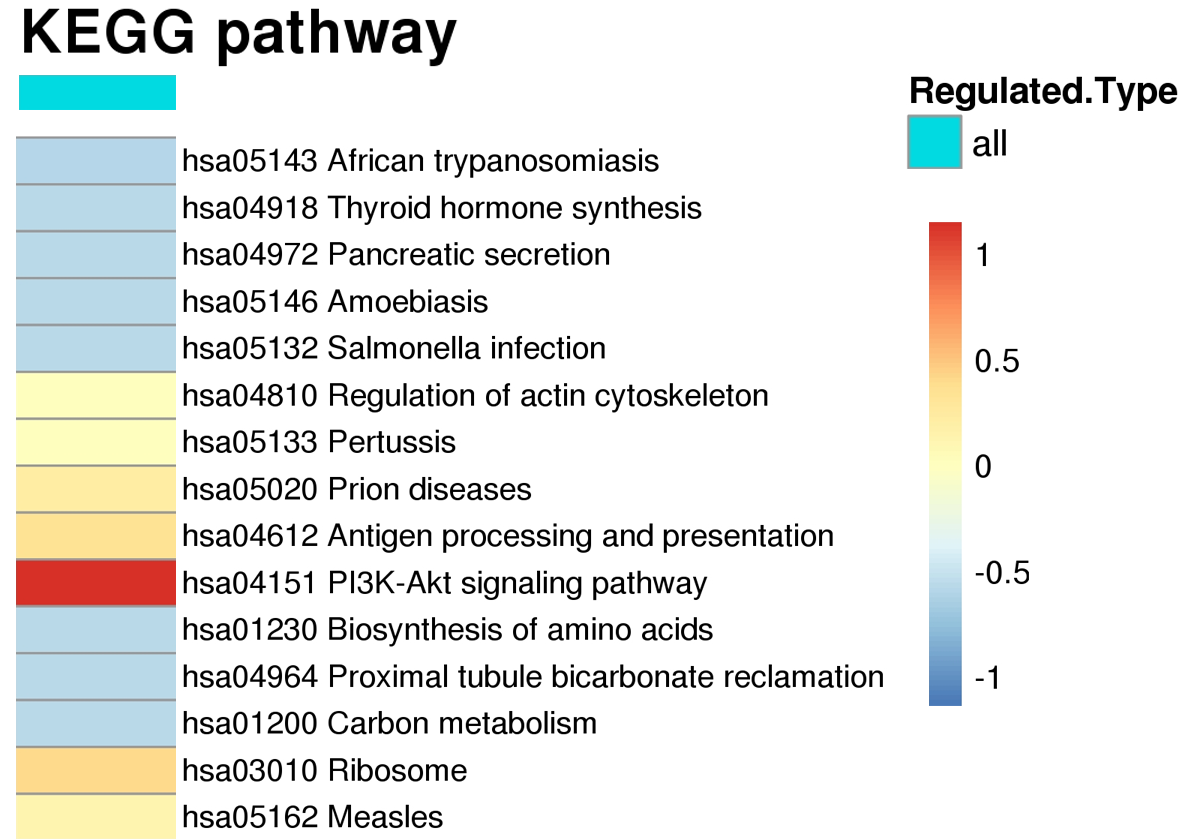

Supplement: Supplementary file 9 — Additional file 9: Fig. S9 KEGG enrichment analysis of the upregulated Khib-modified proteins in the IgAN. [file 12014_2021_9314_MOESM9_ESM.tif]

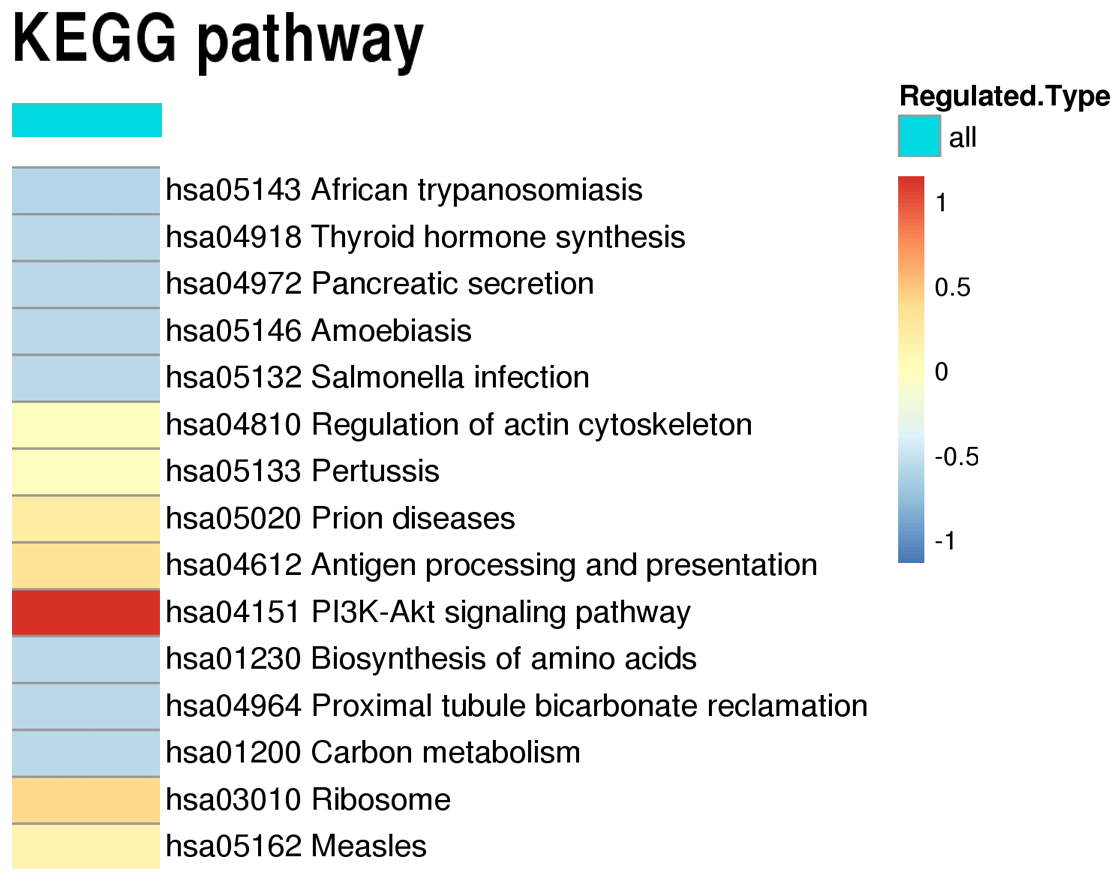

Supplement: Supplementary file 10 — Additional file 10: Fig. S10 KEGG enrichment analysis of the downregulated Khib-modified proteins in the IgAN. [file 12014_2021_9314_MOESM10_ESM.tif]

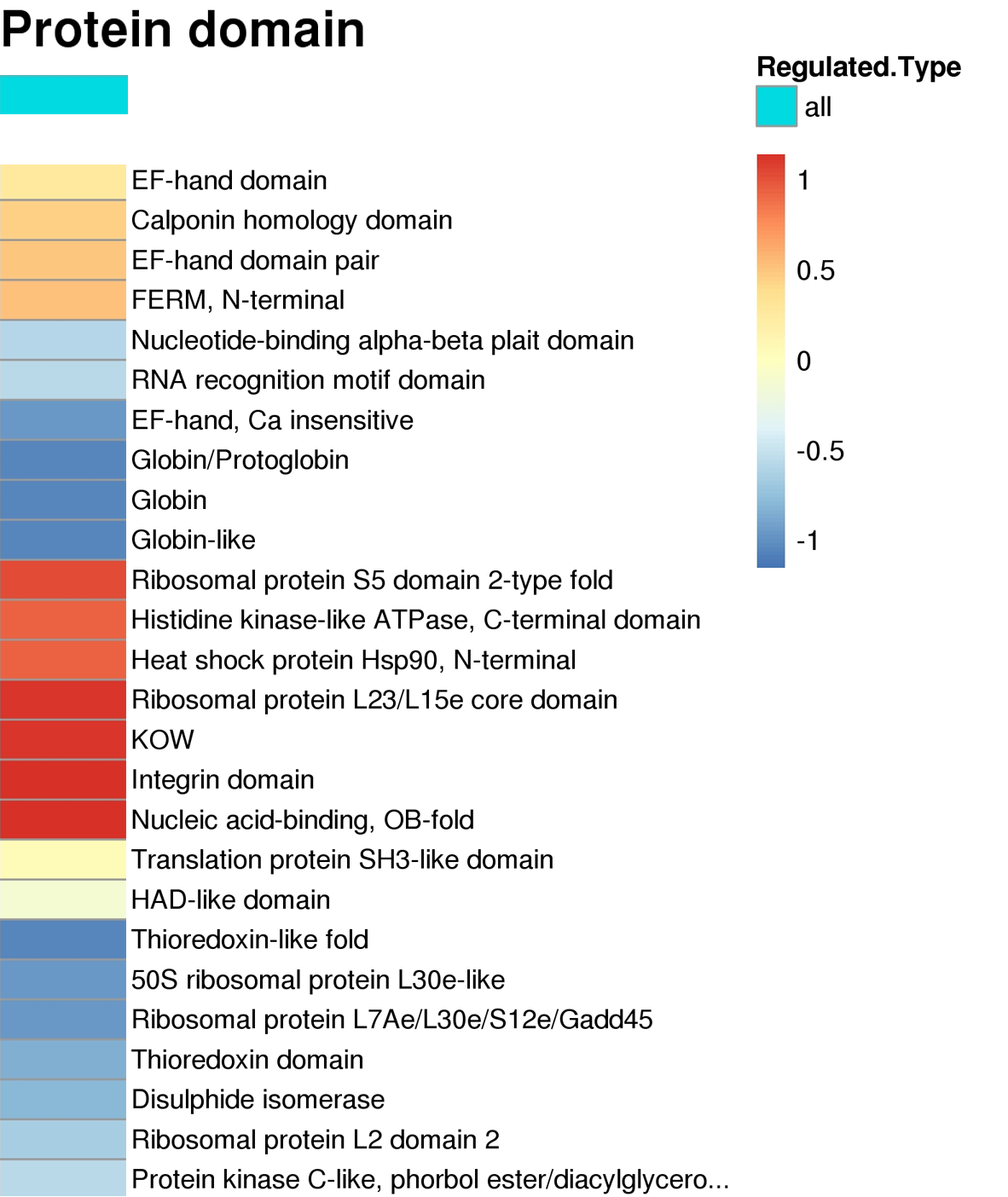

Supplement: Supplementary file 11 — Additional file 11: Fig. S11 Protein domains analysis of the upregulated Khib-modified proteins in the IgAN. [file 12014_2021_9314_MOESM11_ESM.tif]
